# Attenuation of TCR-induced transcription by Bach2 controls regulatory T cell differentiation and homeostasis

**DOI:** 10.1038/s41467-019-14112-2

**Published:** 2020-01-14

**Authors:** Tom Sidwell, Yang Liao, Alexandra L. Garnham, Ajithkumar Vasanthakumar, Renee Gloury, Jonas Blume, Peggy P. Teh, David Chisanga, Christoph Thelemann, Fabian de Labastida Rivera, Christian R. Engwerda, Lynn Corcoran, Kohei Kometani, Tomohiro Kurosaki, Gordon K. Smyth, Wei Shi, Axel Kallies

**Affiliations:** 1 Department of Microbiology and Immunology, The Peter Doherty Institute for Infection and Immunity, The University of Melbourne, Melbourne, VIC 3010 Australia; 2 Molecular Immunology, Walter and Eliza Hall Institute of Medical Research, Melbourne, VIC 3052 Australia; 3 Bioinformatics Division, Walter and Eliza Hall Institute of Medical Research, Melbourne, VIC 3052 Australia; 4 Department of Medical Biology, The University of Melbourne, Melbourne, VIC 3010 Australia; 5 Renal Medicine, Alfred Health, Melbourne, VIC 3004 Australia; 6 Department of Nephrology, Western Health, St Albans, VIC 3021 Australia; 7 QIMR Berghofer Medical Research Institute, Brisbane, QLD 4006 Australia; 8 Laboratory for Lymphocyte Differentiation, RIKEN Center for Integrative Medical Sciences (IMS), Kanagawa, Japan; 9 Laboratory of Lymphocyte Differentiation, WPI Immunology Frontier Research Center, Osaka University, Suita, Osaka 565-0871 Japan; 10 School of Mathematics and Statistics, University of Melbourne, Melbourne, VIC 3010 Australia; 11 School of Computing and Information Systems, The University of Melbourne, Melbourne, VIC 3010 Australia

**Keywords:** Epigenetics in immune cells, Regulatory T cells

## Abstract

Differentiation and homeostasis of Foxp3^+^ regulatory T (Treg) cells are strictly controlled by T-cell receptor (TCR) signals; however, molecular mechanisms that govern these processes are incompletely understood. Here we show that Bach2 is an important regulator of Treg cell differentiation and homeostasis downstream of TCR signaling. Bach2 prevents premature differentiation of fully suppressive effector Treg (eTreg) cells, limits IL-10 production and is required for the development of peripherally induced Treg (pTreg) cells in the gastrointestinal tract. Bach2 attenuates TCR signaling-induced IRF4-dependent Treg cell differentiation. Deletion of IRF4 promotes inducible Treg cell differentiation and rescues pTreg cell differentiation in the absence of Bach2. In turn, loss of Bach2 normalizes eTreg cell differentiation of IRF4-deficient Treg cells. Mechanistically, Bach2 counteracts the DNA-binding activity of IRF4 and limits chromatin accessibility, thereby attenuating IRF4-dependent transcription. Thus, Bach2 balances TCR signaling induced transcriptional activity of IRF4 to maintain homeostasis of thymically-derived and peripherally-derived Treg cells.

## Abstract (web summary)

The transcription factor Bach2 is critical for T cell differentiation, but how it functions in Treg cells is unclear. Here the authors use a Treg-specific mouse model to show that Bach2 controls homeostasis and function of Treg cells by limiting DNA accessibility and activity of IRF4 in response to TCR signaling.

## Introduction

Regulatory T (Treg) cells represent an anti-inflammatory T cell subset that is critical for suppressing autoreactive immune cells^1,2^. The development and function of Treg cells depend on the lineage specific transcription factor Foxp3 and the cytokine interleukin (IL)-2^1,2^. Humans and mice deficient for Foxp3 lack Treg cells and experience multi-organ autoimmune disease due to the resultant immune dysregulation^1,3^. Treg cell development occurs predominantly in the thymus, where high affinity T cell receptor (TCR) interactions and IL-2 signals allow for the stable expression of Foxp3^2,4^. After egress from the thymus, mature Treg cells display a naïve phenotype with high expression of lymphoid homing receptors CD62L and CCR7. Following activation through the TCR, Treg cells can further differentiate into effector (e)Treg cells, which exhibit an activated phenotype and full suppressor function^5,6^. This process requires the coordinated activity of the transcription factors interferon response factor (IRF)-4 and B lymphocyte induced maturation protein (Blimp)1, which together drive the expression of the immunosuppressive cytokine IL-10^7^. Treg cells can be found in many non-lymphoid tissues where they play a crucial role in restraining inflammation and mediating tissue repair and homeostasis^5,6,8^. Tissue Treg cells display an eTreg cell phenotype and require the activity of IRF4 and BATF^7,9–12^, but additional transcriptional regulators drive their further specialization into tissue-specific Treg cells^5,6^.

While the majority of Treg cells develop in the thymus, they may also differentiate from conventional CD4 T cells in the periphery. The main site for peripheral (p)Treg cell differentiation is the gut, where CD4 T cells, in response to microbial or food antigens, can upregulate Foxp3 and contribute locally to immunological homeostasis^13,14^. In addition to Foxp3, these cells also express the retinoic acid-related orphan receptor (ROR)-γt. This contrasts with thymus-derived Treg cells found in the intestinal niche that express the transcription factors GATA3 and Helios^13–16^. TCR signals play a critical and complex role in Treg cell differentiation and maintenance. Treg cell differentiation in the thymus is initiated in response to high affinity TCR signals^17^, and ongoing TCR signaling is required for the maintenance of the mature Treg cell pool^18,19^. In contrast, pTreg cell differentiation is antagonized by strong TCR signals^20–22^, with the TCR responsive kinases AKT and mTORC-1 and mTORC-2 being important inhibitors of Foxp3 induction^17^. While it is clear that TCR signals play a critical role in the development and maintenance of Treg cells, it is poorly understood how TCR signaling-induced differentiation is moderated. This is an important question, as Treg cells show elevated self-reactivity and are consistently exposed to high levels of TCR signaling compared to conventional T cells^23^.

The transcription factor Bach2 plays critical roles in multiple hematopoietic cells, including B and T lineage cells^24,25^. Furthermore, it has been shown to promote the differentiation of Treg cells^26,27^, which was linked to Bach2’s role in repressing genes involved in the differentiation of conventional effector T cells, such as Blimp1 and GATA3. However, it has become clear that Treg cells can express transcription factors associated with T helper (Th) differentiation, including GATA3, T-bet, RORγt or Blimp1, without compromising Foxp3 expression or function^5,6,28,29^, questioning the proposed model of competing effector and regulatory fates. Finally, recent modeling of the transcriptional network underlying tissue Treg cell differentiation identified Bach2 as a potential repressor of tissue-Treg cell differentiation^30^. Thus, multiple lines of evidence indicate that Bach2 plays an important role in Treg cell biology; however, its precise role in Treg cells has remained unclear. To address this question, we deleted Bach2 specifically in Treg cells or in their peripheral CD4^+^ precursors. We found that Bach2 controls the differentiation of eTreg and tissue-resident Treg cells by inhibiting TCR signaling-induced transcriptional programs. Furthermore, Bach2 is required for the development and maintenance of pTreg cells in the gastrointestinal tract. Mechanistically, Bach2 inhibits the genomic binding of IRF4, thereby limiting TCR-driven effector differentiation of Treg cells. Deletion of IRF4 promotes differentiation of induced Treg cells and rescues pTreg cell differentiation in the absence of Bach2. In turn, loss of Bach2 normalizes eTreg cell differentiation of IRF4-deficient Treg cells. Chromatin immunoprecipitation and sequencing (ChIP-seq) and assay for transposase accessible chromatin followed by sequencing (ATAC-seq) showed that Bach2 counteracts the TCR-induced DNA-binding activity of IRF4 and limits chromatin accessibility. Overall, our data indicate that Bach2 is a gatekeeper of Treg cell differentiation that balances the TCR signaling induced transcriptional activity to maintain homeostasis of both thymically and peripherally induced Treg cells.

## Results

### Bach2 limits effector differentiation of mature Treg cells

**Fig. 1:**
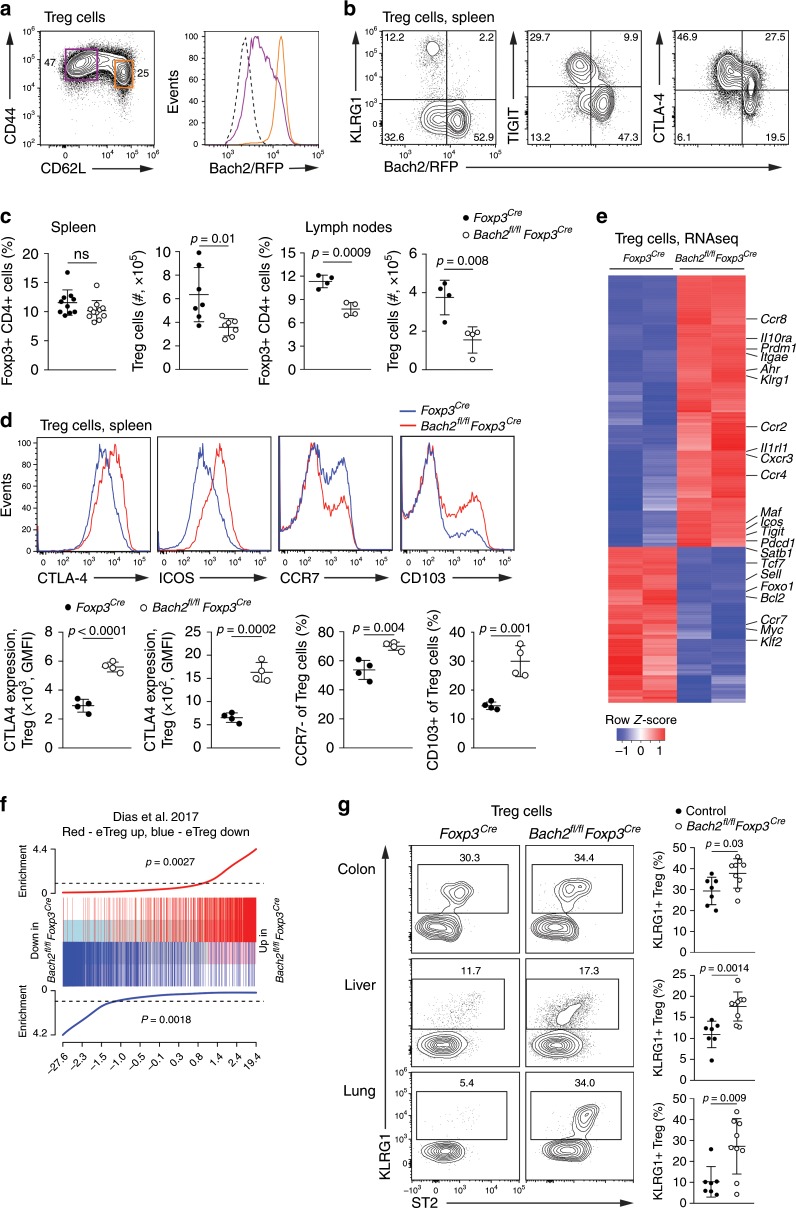
Bach2 limits activation and effector differentiation of mature Treg cells. **a** Flow cytometry plots showing Bach2-RFP reporter expression by splenic Treg cells with naïve (CD62L^+^) and activated (CD62L^-^) phenotypes, or wildtype cells (dashed line). **b** Co-expression of Bach2-RFP with indicated activation-associated molecules. **c** Proportions and numbers and of Treg cells in the spleens and pooled brachial, axial and inguinal lymph nodes of 6 to 8-week-old *Foxp3*^*Cre*^ and *Bach2*^*fl/fl*^*Foxp3*^*Cre*^ mice. **d** Histograms showing expression of indicated molecules (upper) and quantification of their expression (lower), as measured by flow cytometry of splenic Treg cells from 6 to 8-week-old *Foxp3*^*Cre*^ and *Bach2*^*fl/fl*^*Foxp3*^*Cre*^ mice. **e**, **f** Splenic Treg cells from *Foxp3*^*Cre*^ and *Bach2*^*fl/fl*^*Foxp3*^*Cre*^ mice were isolated by flow cytometry and subjected to RNA-seq. **e** Heatmap shows expression of the top 200-most differentially expressed genes, with genes of interest indicated. **f** Gene set enrichment plot for a gene signature of eTreg cells^32^ in the comparison between *Bach2*^*fl/fl*^*Foxp3*^*Cre*^ and control *Foxp3*^*Cre*^ Treg cells. **g** Flow cytometry plots showing expression of KLRG1 and ST2 by Treg cells isolated from the colonic lamina proprium, liver and lung tissue of *Foxp3*^*Cre*^ and *Bach2*^*fl/fl*^*Foxp3*^*Cre*^ mice (left). Frequencies of KLRG1-expressing Treg cells in indicated organs from *Bach2*^*fl/fl*^*Foxp3*^*Cre*^ mice and controls (right). Flow cytometry plots and data in (**a**, **b**, and **d**) are representative of 2–3 independent experiments with at least 6 mice. Data in **c** and **g** are pooled from two independent experiments. Statistical significance was tested using the unpaired Student’s *t-*test. Error bars denote mean ±S.D.; ns – not significant. Source data are provided as a Source Data file.

To map expression of Bach2 within the Treg cell lineage (identified as CD4^+^Foxp3^+^ cells), we utilized a Bach2 reporter mouse line^31^. Expression of Bach2 was highest in CD62L^+^ Treg cells and was partially downregulated in CD62L^−^ Treg cells, suggesting that Bach2 acts in both naïve and activated Treg cells (Fig. 1a). Bach2 expression was lowest in eTreg cells identified by expression of the maturation marker killer cell lectin-like receptor subfamily G1 (KLRG1), the inhibitory receptor T cell immunoreceptor with Ig and ITIM domains (TIGIT), cytotoxic T lymphocyte associated antigen (CTLA)-4 (Fig. 1b) and Blimp1 (Supplementary Fig. 1a). To examine the role of Bach2 in Treg cells, we crossed mice carrying floxed Bach2 alleles with *Foxp3*^*Cre*^ mice, resulting in deletion of Bach2 in mature Treg cells. *Bach2*^*fl/fl*^*Foxp3*^*Cre*^ mice appeared healthy and did not show any impaired survival or obvious signs of autoimmune pathology compared to *Foxp3*^*Cre*^ control mice (Supplementary Fig. 1b). Similarly, we detected no increase in activated conventional T cells in *Bach2*^*fl/fl*^*Foxp3*^*Cre*^ mice compared with controls (Supplementary Fig. 1c). *Bach2*^*fl/fl*^*Foxp3*^*Cre*^ mice had significantly reduced Treg cell numbers in peripheral lymph nodes compared with *Foxp3*^*Cre*^ control mice (Fig. 1c, Supplementary Fig. 1d). Similar results were obtained in *Bach2*^*fl/fl*^*Cd4*^*Cre*^ mice, in which Bach2 is deleted from all T cells prior to Treg lineage commitment (Supplementary Fig. 1e). Notably, we observed substantial activation of Bach2-deficient Treg cells in comparison to control Treg cells in *Bach2*^*fl/fl*^*Foxp3*^*Cre*^ mice, with increased expression of markers associated with eTreg cell differentiation, including CTLA-4, the inducible costimulator (ICOS), the αE integrin CD103, and reduced expression of the lymphoid homing receptor CCR7, usually expressed by naïve Treg cells (Fig. 1d). To broadly examine the impact of Bach2 on Treg cells, we performed RNA sequencing (RNA-seq) of Treg cells isolated by flow cytometry from the spleens of *Bach2*^*fl/fl*^*Foxp3*^*Cre*^ and *Foxp3*^*Cre*^ control mice. In total, we detected 1207 genes differentially expressed (FDR < 0.05) between Bach2-deficient and control Treg cells. Notably, many genes involved in eTreg cell differentiation, such as *Prdm1* (encoding Blimp1, from here on *Blimp1*), *Il1rl1* (encoding the IL-33 receptor ST2), *Il10*, *Il10ra*, *Tigit*, *Icos*, *Maf*, *Ccr2* and *Ccr4*, were upregulated in Bach2-deficient Treg cells, while genes associated with the naïve state, including *Ccr7*, *Sell*, *Foxo1*, *Tcf7* (encoding TCF1), *Klf2* and *Satb1*, were downregulated (Fig. 1e, Supplementary Fig. 1f). Indeed, a gene set enrichment test using a previously published signature of eTreg cells^32^ showed that genes upregulated during eTreg cell differentiation were enriched in Bach2-deficient Treg cells, while genes downregulated during eTreg differentiation were enriched in control Treg cells (Fig. 1f). Consistent with the idea that Bach2 is a negative regulator of eTreg cell differentiation, *Bach2*^*fl/fl*^*Foxp3*^*Cre*^ compared with control mice had elevated numbers of tissue Treg cells, identified by KLRG1 expression, in non-lymphoid tissues such as the colon lamina propria, liver and lung (Fig. 1g). Together, these observations suggest that Bach2 acts in naïve and early activated Treg cells to prevent premature activation and eTreg cell differentiation.

### Bach2 is required for of Treg cell expansion and maintenance

**Fig. 2:**
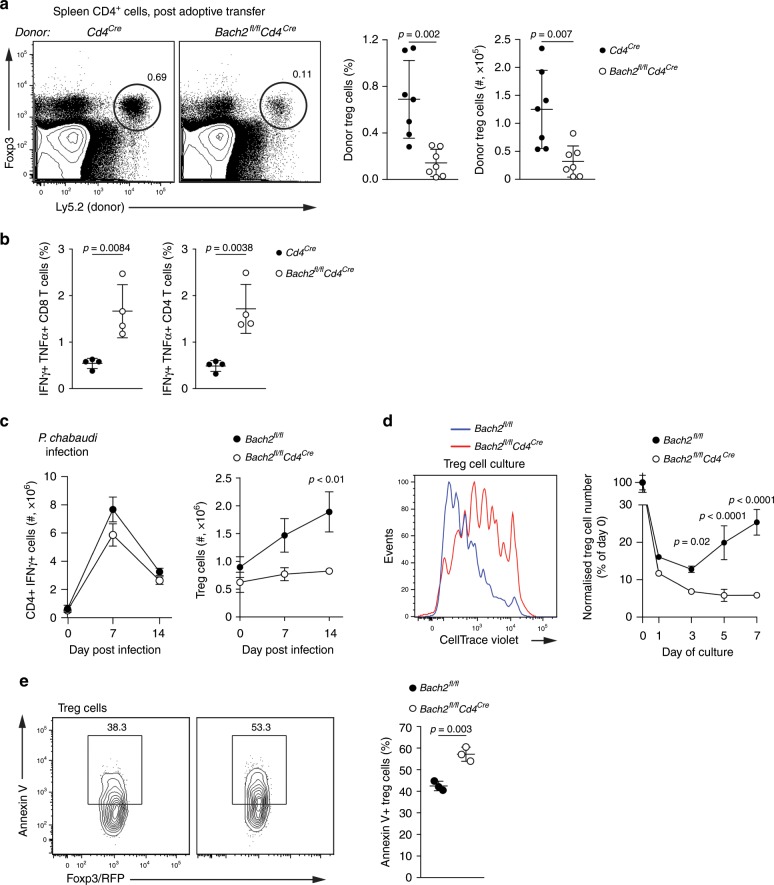
Bach2 is required for the survival and proliferation of Treg cells. **a**, **b** Treg cells sorted from *Cd4*^*Cre*^ or *Bach2*^*fl/fl*^*Cd4*^*Cre*^ mice were adoptively transferred into congenically marked Foxp3 diphtheria toxin receptor transgenic mice depleted of endogenous Treg cells by injection of diphtheria toxin. **a** Representative flow cytometry plots indicate the frequency of donor Treg cells among the CD4^+^ compartment (left), and quantification of Treg cell numbers (right), 7 days following adoptive transfer. **b** Frequency of IFN-γ/TNF co-expressing cells among the splenic CD8 and conventional CD4 T cell populations, as measured by flow cytometry. **c** Splenic Th1 (left) and Treg (right) cell numbers from *Bach2*^*fl/fl*^*Cd4*^*Cre*^ and *Bach2*^*fl/fl*^ control mice following infection with *Plasmodium chabaudi*. **d** Flow cytometry histograms of CellTrace Violet dilution at day 5 of culture (left) and normalized total cell numbers of Treg cells from *Bach2*^*fl/fl*^*Cd4*^*Cre*^ and *Bach2*^*fl/fl*^ control mice at the indicated culture time points (right). **e** Cell-surface binding of Annexin V by Treg cells isolated from *Bach2*^*fl/fl*^ and *Bach2*^*fl/fl*^*Cd4*^*Cre*^ mice following 24 h of culture. Flow cytometry plots and data from (**b**–**d**) are representative, data in **a** are pooled from two independent experiments with at least four mice per genotype. Significance tested using two-way ANOVA with the Šidák correction for multiple comparisons (**d**). Otherwise, statistical significance was tested using the unpaired Student’s *t-*test. Error bars denote mean ±S.D. **a**, **b**, **d**, **e** or ±S.E.M. **c** Source data are provided as a Source Data file.

To test the ability of Bach2-deficient Treg cells to expand in response to inflammatory signals, we used an adoptive transfer model. We depleted Treg cells from Foxp3-diphteria toxin receptor transgenic mice^33^ and adoptively transferred into these mice small numbers of Bach2-deficient or control Treg cells. 1 week following transfer and deletion of endogenous Treg cells, Bach2-deficient Treg cells were significantly reduced compared to control Treg cells (Fig. 2a), indicating that Bach2 is required for proliferation and/or survival of Treg cells. Consistent with this notion, the spleens of mice that received Bach2-deficient Treg cells contained increased numbers of IFN-γ and TNF expressing conventional T cells compared with recipients of control Treg cells (Fig. 2b). To assess whether Bach2 is required for the expansion of Treg cells in response to an infectious challenge known to induce proliferation of both conventional effector T cells and Treg cells^34,35^, we infected *Bach2*^*fl/fl*^*Cd4*^*Cre*^ and control mice with *Plasmodium chabaudi*. Following infection, we observed a substantial expansion of IFN-γ^+^ CD4 effector T cells in both control and *Bach2*^*fl/fl*^*Cd4*^*Cre*^ mice (Fig. 2c, left). In contrast, Treg cells expanded in the spleens of control but not *Bach2*^*fl/fl*^*Cd4*^*Cre*^ mice (Fig. 2c, right). To directly assess the impact of Bach2 on the survival and proliferation of Treg cells in vitro, we sorted *Foxp3*^*RFP*^-positive Treg cells from *Bach2*^*fl/fl*^*Cd4*^*Cre*^ and control mice, cultured them in the presence of αCD3 antibody and IL-2, and measured cell division and numbers. Bach2-deficient Treg cells displayed a defect in proliferation as measured by dilution of the division tracking dye CellTrace Violet and failed to expand compared with Bach2-sufficient control Treg cells (Fig. 2d). Consistent with these findings, we observed significantly more binding of Annexin V to Bach2-deficient Treg cells following 24 h in culture, indicating increased apoptosis in the absence of Bach2 (Fig. 2e). In line with this conclusion, expression of prosurvival *Bcl2* was significantly lower in Bach2-deficient compared to control Treg cells (Fig. 1e). Overall these results indicate a requirement for Bach2 to support the survival and expansion of the regulatory T cell compartment.

### Bach2 restrains Treg cell suppressive function in colitis

**Fig. 3:**
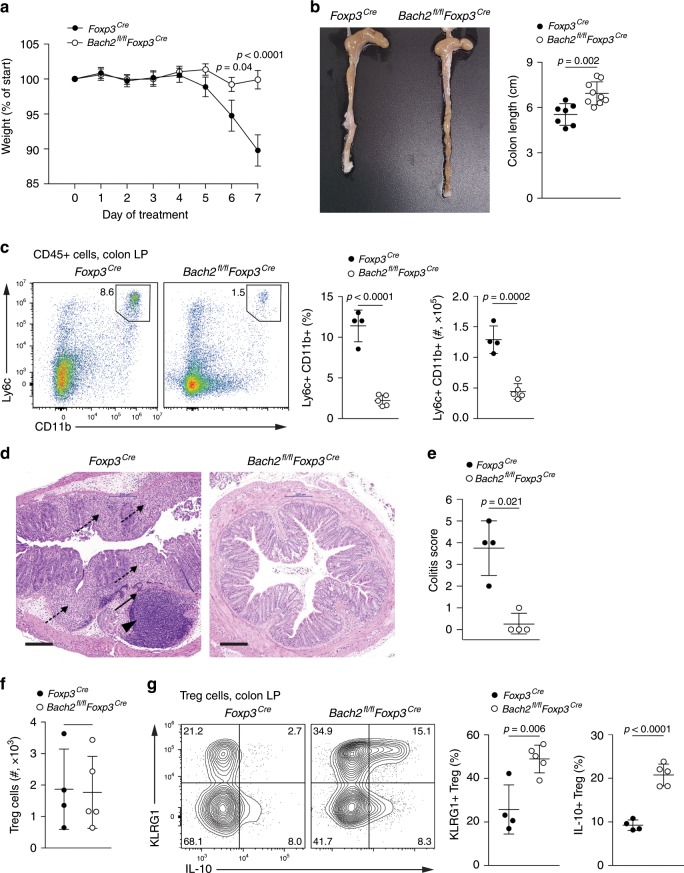
Bach2 restrains Treg cell suppressive function in colitis. *Bach2*^*fl/fl*^*Foxp3*^*Cre*^ and *Foxp3*^*Cre*^ control mice were administered 1.5% (w/v) dextran sodium sulfate (DSS) in drinking water for 5 days and regular drinking water for another two days. **a** Mouse weight over time normalized to starting weight. **b** Representative colons from DSS-treated *Foxp3*^*Cre*^ and *Bach2*^*fl/fl*^*Foxp3*^*Cre*^ mice (left) and quantification of colon length (right). **c** Flow cytometry plots quantifying monocytes in the colonic lamina propria. **d** Histological staining of the distal colon of DSS-treated mice. Arrowhead indicates submucosal inflammatory infiltrate, solid arrow, transmural inflammatory infiltrate, dashed arrow, mucosal inflammatory infiltrate and erosion of the crypt-villus architecture. Scale bar = 200 μm. **e** Quantification of disease scores between genotypes. **f** Frequencies of Treg cells in the colonic lamina propria of DSS-treated *Foxp3*^*Cre*^ and *Bach2*^*fl/fl*^*Foxp3*^*Cre*^ mice. **g** Flow cytometry plots showing IL-10 and KLRG1 expression by Treg cells from the lamina propria (left), and quantification of expression (right). Flow cytometry plots are representative, data are pooled from (**a** and **b**) or representative of (**c**–**g**) two independent experiments with seven control and nine conditional knockout mice. Significance tested using two-way ANOVA with Šidák correction for multiple comparisons (**a**). Otherwise, statistical significance was tested using the unpaired Student’s *t-*test. Error bars denote mean ± S.D.; ns–not significant. Source data are provided as a Source Data file.

Our data indicate that Bach2-deficient Treg cells show increased ability to differentiate into tissue Treg cells (Fig. 1g). To test the functional consequences of Bach2 loss in non-lymphoid tissue inflammation, we utilized a model of chemically induced colitis, which is controlled by Treg cells in a manner dependent on the immunomodulatory cytokine IL-10 (refs. ^36–38^). We administered *Bach2*^*fl/fl*^*Foxp3*^*Cre*^ and *Foxp3*^*Cre*^ control mice dextran sodium sulfate (DSS) dissolved in drinking water and assessed the resulting colitis. As expected, *Foxp3*^*Cre*^ control mice displayed severe disease including weight loss, colon shortening, monocytic colon infiltrate and distal colon tissue destruction (Fig. 3a–d). In contrast, mice with Bach2-deficient Treg cells were largely protected from colitis and showed an overall reduced disease score compared with control mice (Fig. 3a–e), suggesting protection due to improved Treg cell function in the absence of Bach2. We did not observe any differences in the overall numbers of Treg cells recovered from the colonic lamina propria of DSS-treated mice (Fig. 3f). However, consistent with Bach2 limiting eTreg and tissue Treg cell differentiation, *Bach2*^*fl/fl*^*Foxp3*^*Cre*^ mice showed substantially increased frequencies of colonic Treg cells expressing KLRG1 and IL-10 compared with Treg cells from control mice (Fig. 3g). Together, these observations indicate that Bach2 restricts the activation and effector differentiation of Treg cells during the response to inflammation in peripheral tissues.

### pTreg cell differentiation intrinsically requires Bach2

**Fig. 4:**
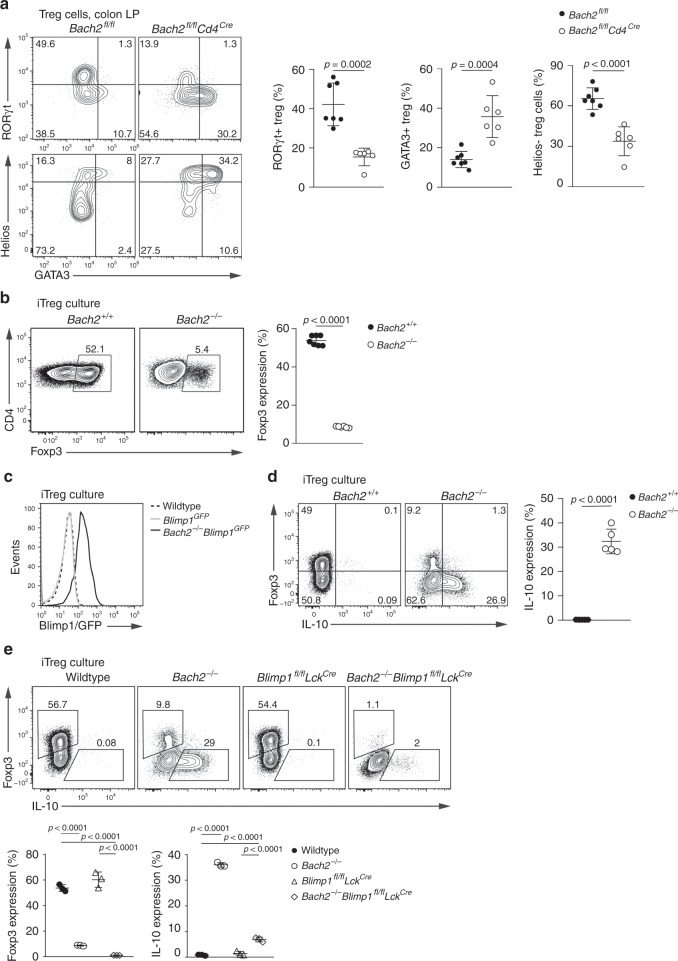
Bach2 is intrinsically required for pTreg cell differentiation. **a** Flow cytometry plots and quantification showing GATA3, RORγt and Helios expression by Treg cells from the colonic lamina propria of *Bach2*^*fl/fl*^*Cd4*^*Cre*^ and control mice. **b** Flow cytometry plots showing Foxp3 expression by wildtype and *Bach2*^*−/−*^ CD4 T cells after three days in Treg cell-inducing culture conditions, and frequency of Foxp3-expressing cells (right). **c** Histograms showing Blimp1-GFP expression by *Blimp1*^*GFP*^ and *Bach2*^*−/−*^*Blimp1*^*GFP*^ CD4 T cells after culture in Treg cell-inducing conditions. Dashed line indicates background fluorescence levels in non-reporter cells. **d** Flow cytometry plots showing Foxp3 and IL-10 expression by wildtype and *Bach2*^*−/−*^ CD4 T cells after Treg cell-inducing culture, and quantification (right). **e** Flow cytometry plots showing Foxp3 expression by wildtype, *Bach2*^*−/−*^, *Blimp1*^*fl/fl*^*Lck*^*Cre*^ and *Bach2*^*−/−*^*Blimp1*^*fl/fl*^*Lck*^*Cre*^ after three days culture in Treg cell-inducing conditions, and frequency of Foxp3 and IL-10 expressing cells (below). Flow cytometry plots are representative, data pooled from (**a**) or representative of two (**c**–**e**) or six (**b**) independent experiments. Statistical significance tested using two-way ANOVA with Tukey’s test (**e**). Otherwise, significance was tested using the unpaired Student’s *t-*test. Error bars denote mean ± S.D. Source data are provided as a Source Data file.

Intestinal Treg cells derive from two distinct developmental pathways. Thymically derived intestinal Treg cells express the transcription factors GATA3 and Helios, while Treg cells induced peripherally from conventional T cells (pTreg cells) express the transcription factor RORγt and lack Helios (reviewed in ref. ^14^). Notably, both in DSS colitis and in steady state, we observed reduced numbers of intestinal RORγt^+^ Treg cells in *Bach2*^*fl/fl*^*Foxp3*^*Cre*^ mice, suggesting that Bach2 plays a role in maintaining pTreg cells (Supplementary Fig. 2a, b). In order to study the role of Bach2 in pTreg cell differentiation, we used *Bach2*^*fl/fl*^*Cd4*^*Cre*^ mice, in which T cells lose Bach2 expression prior to Foxp3 expression. Strikingly, *Bach2*^*fl/fl*^*Cd4*^*Cre*^ mice showed a severe reduction in RORγt^+^ colonic Treg cells, whereas GATA3^+^ Treg cell frequencies were increased compared to control animals (Fig. 4a, upper). Similarly, colons of *Bach2*^*fl/fl*^*Cd4*^*Cre*^ mice showed a significantly reduced frequency of Helios-negative Treg cells compared with controls (Fig. 4a, lower), indicating an overall reduction of the pTreg cell compartment, rather than a loss of RORγt expression. Notably, we did not observe differences in RORγt expression by colonic conventional CD4 T cells from *Bach2*^*fl/fl*^*Cd4*^*Cre*^ and control mice (Supplementary Fig. 2c). These results show that Bach2 is required for the development and maintenance of intestinal pTreg cells.

To test the role for Bach2 in the generation of pTreg cells, we utilized an in vitro model of pTreg cell development using TCR stimulation of naïve splenic conventional CD4 T cells in the presence of IL-2 and transforming growth factor (TGF)-β^39^. Consistent with published data^26,27^, significantly fewer Bach2-deficient cells upregulated Foxp3 compared with controls (Fig. 4b). Premature expression of transcription factors, including Blimp1 and GATA3, that drive effector T cell differentiation was proposed to be responsible for impaired pTreg differentiation in the absence of Bach2 (refs. ^26,27^). Indeed, we observed unrestricted expression of Blimp1 (Fig. 4c) and substantially increased production of IL-10, a downstream target of Blimp1^7^, in Bach2-deficient inducible Treg cell cultures (Fig. 4c) and various T helper (Th) cell polarizing culture conditions **(**Supplementary Fig. 3a, b). To test whether premature Blimp1 expression was responsible for the block in Treg cell differentiation in the absence of Bach2, we generated *Bach2*^*−/−*^*Blimp1*^*fl/fl*^*Lck*^*Cre*^ mice that lack Bach2 and Blimp1 in T cells. However, impaired induction of Foxp3 was independent of Blimp1, as Bach2/Blimp1 double-deficient CD4 T cells showed a similar block in Treg differentiation as cells lacking Bach2 only (Fig. 4e). This phenotype was also independent of the use of antibodies blocking the activity of the pro-inflammatory cytokines IL-4 and IFN-γ, which reduced GATA3 expression in Bach2-deficient cultures but did not rescue Treg differentiation in vitro (Supplementary Fig. 3c). These results suggested that premature acquisition of a conventional effector T cell phenotype was not causative for the lack of Treg cell differentiation by Bach2 deficient cells, and that Bach2 itself is required for pTreg cell differentiation.

### Bach2 limits TCR responsiveness

**Fig. 5:**
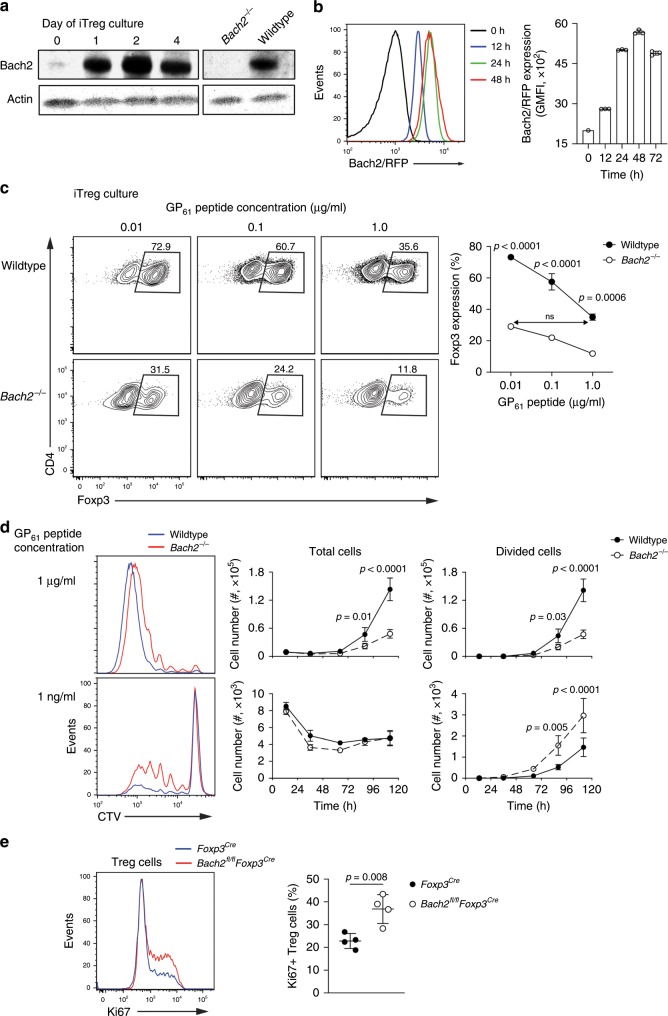
Bach2 supports pTreg cell differentiation by limiting sensitivity to TCR signals. **a** Expression kinetics of Bach2 protein measured by Western blot of sorted naïve CD62L^+^ CD4 T cells cultured in Treg cell inducing conditions for up to 4 days. **b** Representative histograms (left) and quantification (right) of expression of Bach2-RFP reporter following stimulation of naïve CD4 T cells from *Bach2*^*RFP*^ mice isolated as above, as measured by flow cytometry at the indicated time points following culture. **c** Flow cytometry plots showing Foxp3 expression by splenic naïve CD4 T cells from congenically marked wildtype and *Bach2*^*−/−*^ Smarta TCR transgenic mice, cocultured for three days in Treg cell-inducing culture conditions with indicated concentrations of antigenic GP_61_ peptide, and frequency of Foxp3 expressing cells (right). **d** Histograms showing CellTrace Violet dilution at day 5 of culture of splenic naïve CD4 T cells from wildtype and *Bach2*^*−/−*^ Smarta mice cultured with the indicated concentrations of GP_61_ peptide (left), and quantification of the number of total cells and of divided cells in the cultures (right). **e** Histogram showing Ki67 expression (left) and quantification (right), as measured by flow cytometry of Treg cells from pooled brachial, axial and inguinal lymph nodes from *Foxp3*^*Cre*^ and *Bach2*^*fl/fl*^*Foxp3*^*Cre*^ mice. Data are representative of two independent experiments. Significance tested using two-way ANOVA with Tukey’s test for multiple comparisons (**c**, **d**). Otherwise, significance was tested using the unpaired Student’s *t-*test. Error bars denote mean ± S.D.; ns – not significant. Source data are provided as a Source Data file.

Induced Treg cell development is controlled by TCR signaling^20,21^. To examine the expression of Bach2 during T cell activation we made use of a new Bach2 antibody and Bach2 reporter mice. Bach2 was expressed by naïve CD4 T cells (Supplementary Fig. 4a); however, TCR signals substantially increased its expression (Fig. 5a, b), suggesting that it mainly acts in activated T cells. To examine the role of TCR signals in more detail, we generated Bach2-deficient Smarta TCR transgenic mice, which harbor CD4 T cells specific for a peptide comprising amino acid residues 61–80 of the glycoprotein (GP_61_) of the lymphocytic choriomeningitis virus. We then co-cultured congenically marked naïve CD4 T cells from control and Bach2-deficient Smarta TCR transgenic mice together with a range of concentrations of GP_61_ peptide in Treg-inducing culture conditions. As expected, Foxp3 expression increased with reduced peptide concentrations (Fig. 5c, upper) and a significantly smaller proportion of the Bach2-deficient T cells expressed Foxp3 compared with co-cultured control T cells (Fig. 5c, lower). Notably, however, Foxp3 expression was similar when comparing *Bach2*^*−/−*^ T cells stimulated in the presence of low amounts of peptide (0.01 μg/mL peptide) and wildtype control T cells stimulated in the presence of high amounts of peptide (1.0 μg/mL) (Fig. 5c, right), revealing an approximately 100-fold difference in TCR-dependent induction of Foxp3 expression directly attributable to Bach2. Consistent with a lower threshold for TCR-induced activation in the absence of Bach2, a larger number of Bach2-deficient Smarta-transgenic T cells proliferated in response to low amounts of GP_61_ peptide compared to co-cultured wildtype cells (Fig. 5d). In line with these results, we observed a higher frequency of proliferating Treg cells, as measured by Ki67 expression, in the lymphoid organs of *Bach2*^*fl/fl*^*Foxp3*^*Cre*^ compared to *Foxp3*^*Cre*^ control mice (Fig. 5e). Together, these data suggest that Bach2 controls Treg cell differentiation by limiting sensitivity to TCR signals.

### IRF4 loss rescues Bach2-deficient Treg cell differentiation

**Fig. 6:**
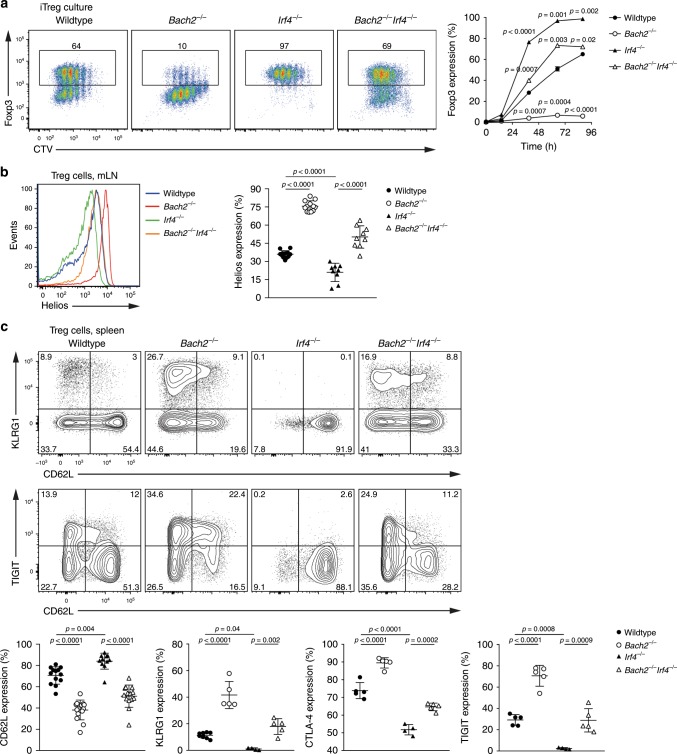
Loss of IRF4 rescues pTreg and eTreg cell differentiation in the absence of Bach2. **a** Flow cytometry plots showing Foxp3 expression by CD4 T cells from wildtype, *Bach2*^*−/−*^, *Irf4*^*−/−*^, and *Bach2*^*−/−*^*Irf4*^*−/−*^ mice following culture in Treg cell-inducing conditions, and frequency of Foxp3 expressing cells at the indicated times post culture (right). **b** Histograms of Helios expression by Treg cells from the mesenteric lymph nodes of wildtype, *Bach2*^*−/−*^, *Irf4*^*−/−*^, and *Bach2*^*−/−*^*Irf4*^*−/−*^ mice (left), and frequency of Helios-expressing Treg cells (right). **c** Flow cytometry plots showing expression of indicated molecules by splenic Treg cells from wildtype, *Bach2*^*−/−*^, *Irf4*^*−/−*^, and *Bach2*^*−/−*^*Irf4*^*−/−*^ mice, and quantification of indicated molecules (below). Significance tested using two-way ANOVA with Dunnett’s test for multiple comparisons (**a**) or using ordinary one-way ANOVA with Tukey’s test for multiple comparisons (**c**). Data are representative of (**a**) or pooled (**b**, **c**) from three independent experiments. Error bars denote mean ± S.D. Source data are provided as a Source Data file.

IRF4 is a transcription factor that is induced by TCR signals and mediates TCR-dependent transcription in a dose-dependent manner^40^. To explore the impact of TCR-dependent transcription on Treg cell development, we generated Bach2/IRF4-double deficient (*Bach2*^*−/−*^*Irf4*^*−/−*^) mice. We then cultured naïve CD4 T cells isolated from *Bach2*^*−/−*^, *Irf4*^*−/−*^, *Bach2*^*−/−*^*Irf4*^*−/−*^ or wildtype control mice in Treg cell-inducing conditions. Consistent with the notion that attenuated TCR signals promote inducible Treg cell differentiation, a much larger proportion of *Irf4*^*−/−*^ cells expressed Foxp3 compared to wildtype T cells, while the majority of *Bach2*^*−/−*^ cells did not express Foxp3 (Fig. 6a). Strikingly, additional loss of IRF4 restored Foxp3 expression to Bach2-deficient cells, with an even greater proportion of *Bach2*^*−/−*^*Irf4*^*−/−*^ cells expressing Foxp3 than wildtype controls (Fig. 6a). Similar results were obtained with cells from mice in which Bach2 and IRF4 were specifically deleted from T cells (*Bach2*^*fl/fl*^*Irf4*^*fl/fl*^*Cd4*^*Cre*^) (Supplementary Fig. 4b).

In order to assess pTreg cell differentiation in vivo, we analyzed the intestinal compartments of wildtype, *Bach2*^*−/−*^, *Irf4*^*−/−*^, and *Bach2*^*−/−*^*Irf4*^*−/−*^ mice. We were unable to isolate Treg cells in sufficient quantities for analysis from the intestinal lamina propria of *Irf4*^*−/−*^ and *Bach2*^*−/−*^*Irf4*^*−/−*^ mice, which is in line with the central role of IRF4 in tissue Treg cell differentiation and RORγt expression (Supplementary Fig. 4c)^7,10,41^. However, consistent with a negative role of IRF4 in pTreg cell generation, analysis of mesenteric lymph nodes of IRF4-deficient mice showed increased proportions of Helios-negative Treg cells compared to wildtype mice, and loss of IRF4 partially normalized the increased Helios expression seen in Bach2-deficient Treg cells (Fig. 6b).

IRF4 plays a critical role in the differentiation of eTreg cells^7^. Accordingly, *Irf4*^*−/−*^ Treg cells were largely CD62L^+^ and lacked TIGIT, CTLA4, and KLRG1 expression (Fig. 6c). Strikingly, however, additional loss of Bach2 in *Irf4*^*−/−*^ Treg cells resulted in rescue of expression of TIGIT, KLRG1, CTLA4, and CD62L (Fig. 6c). Restoration of activated IRF4-deficient Treg cells by additional loss of Bach2 was also observed in mice in which Bach2 and IRF4 were specifically deleted from T cells (*Bach2*^*fl/fl*^*Irf4*^*fl/fl*^*Cd4*^*Cre*^), indicating the T cell intrinsic nature of this phenotype (Supplementary Fig. 4d). Overall, these observations show that in response to TCR signaling, Bach2 and IRF4 act in an opposing manner to direct both pTreg and eTreg cell differentiation.

### Increased IRF4-dependent transcription without Bach2

**Fig. 7:**
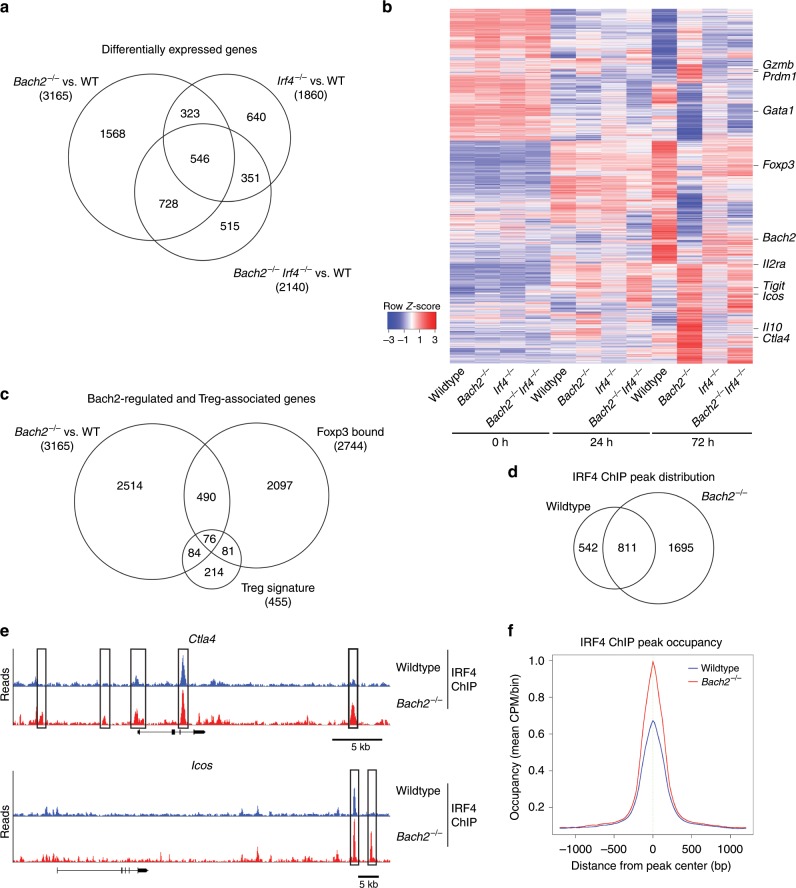
IRF4-dependent transcriptional deregulation in the absence of Bach2. **a–c** Naive (CD62L^+^CD44^−^CD25^−^) splenic CD4 T cells from wildtype, *Bach2*^*−/−*^, *Irf4*^*−/−*^ and *Bach2*^*−/−*^*Irf4*^*−/−*^ mice were cultured in Treg cell-inducing conditions for 0 h, 24 h, or 72 h and subjected to RNA-seq. Numbers of genes significantly differentially expressed between wildtype and each knockout genotype at 72 h (**a**). Heatmap showing expression (*Z*-scores) of genes differentially expressed in *Bach2*^*−/−*^ compared to wildtype cells at each analysis time point (**b**). Overlap between genes differentially expressed in the 72 h *Bach2*^*−/−*^ vs. wildtype comparison, and those previously identified either as Foxp3-bound^11^ or as Treg cell signature genes^42^ (**c**). **d**–**f** ChIP-seq for IRF4 performed on naïve splenic CD4 T cells from wildtype and *Bach2*^*−/−*^ mice cultured in Treg cell-inducing conditions for 72 h. Numbers of peaks identified in each genotype (**d**), and example tracks showing loci with *de novo* or enhanced IRF4 binding in the absence of Bach2 (**e**). Mean occupancy of IRF4 at all called IRF4 ChIP peaks in wildtype and *Bach2*^*−/−*^ cells (**f**). Source data are provided as a Source Data file.

To identify the mechanisms by which Bach2 and IRF4 control pTreg cell differentiation, we performed transcriptional profiling by RNA-seq of CD4 T cells from *Bach2*^*−/−*^, *Irf4*^*−/−*^, *Bach2*^*−/−*^*Irf4*^*−/−*^ and wildtype mice, before, after 24 h, and after 72 h of culture in Treg cell-inducing conditions. While naïve T cells from either genotype were similar, substantial differential gene expression was detected at 24 h and even more pronounced at 72 h of culture. Bach2-deficient T cells showed broadly deregulated gene expression, with a total of 3165 genes differentially expressed (>2 fold, 0.05 FDR) compared to wildtype T cells at 72 h of culture (Fig. 7a, b). In contrast, only 2140 genes were differentially expressed when comparing *Bach2*^*−/−*^*Irf4*^*−/−*^ and wildtype cells, while expression of >60% of the genes deregulated in the absence of Bach2 was normalized with additional loss of IRF4 (Fig. 7a, b). Genes deregulated in the absence of Bach2 included eTreg signature genes such as *GzmB*, *Tigit*, *Icos*, *Il10*, and *Ctla4* but also chromatin modifiers such as *Crebp*, *Ep300*, *Kmt2a*, *Ezh1*, *Tet2*, *Dnmt3b*, *Set*, and *Setd1b*, many of which were normalized in their expression in the combined absence of IRF4 and Bach2 (Fig. 7b). As Bach2-deficient T cells were severely impaired in upregulating Foxp3, we compared our data to published lists of Treg cell signature^42^ and Foxp3 target^11^ genes. Importantly, only a small fraction (160 genes, or 5.1%) of the genes deregulated in *Bach2*^*−/−*^ T cells were Treg cell signature genes, including *Foxp3, Cd38, Eno3, Itih5, Cnga1*, *Cd83*, and *Gata1*, or were direct Foxp3 targets (568 genes, or 17.9%), including *Ccr4*, *Pdcd1*, *Tigit*, *Icos*, *Myb*, and *Nrp1* (Fig. 7c). Consistent with this observation, the pattern of deregulated gene expression in the absence of Bach2, and their normalization with the additional loss of IRF4, was intact independent from Foxp3 binding or inclusion into the Treg cell signature (Supplementary Fig. 5). These results indicate that the IRF4-dependent rescue of deregulated gene expression and block in Treg cell differentiation induced by Bach2 loss is independent of the activity of Foxp3 itself.

We next assessed the effect of Bach2 loss on the genomic binding of IRF4 by ChIP-seq using anti-IRF4 antibodies. We identified binding of IRF4 at 1353 sites associated with 1208 genes. This included multiple genes previously reported to be bound by IRF4 such as *Prdm1, Ctla4*, *Icos*, and *Il7r* ^10,40^. Strikingly, when we performed IRF4 ChIP-seq on *Bach2*^*−/−*^ CD4 T cells cultured in Treg cell-inducing conditions, we identified binding of IRF4 to 2506 sites (associated with 1929 genes), almost twice the number identified in wildtype (Fig. 7d), including genes transcriptionally deregulated in the absence of Bach2, such as *Ctla4* and *Icos* (Fig. 7e). Indeed, analysis of total IRF4 binding sites showed substantial enrichment of IRF4 binding in Bach2-deficient compared to wildtype cells (Fig. 7f). Overall these results suggest that Bach2 regulates transcription by limiting binding of IRF4 to target genes.

### Bach2 limits DNA accessibility and IRF4 occupancy

**Fig. 8:**
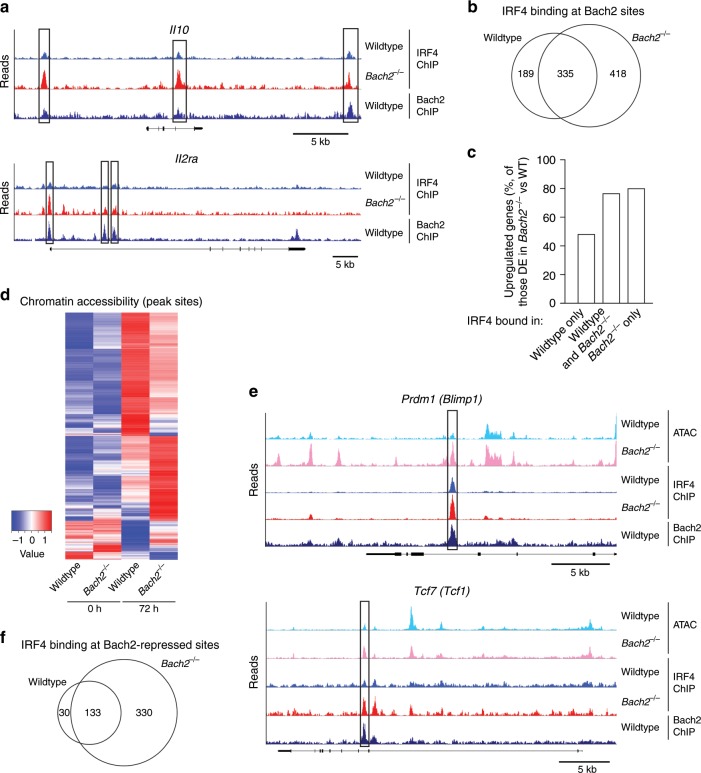
Bach2 limits DNA accessibility, occupancy and expression of IRF4 target genes. **a** Bach2 and IRF4 ChIP-seq tracks showing sites of Bach2 and IRF4 co-binding within the *Il10* and *Il2ra* loci of naïve splenic CD4 T cells cultured in Treg cell-inducing conditions. Boxes indicate Bach2 binding sites with increased IRF4 occupancy in the absence of Bach2. **b** Quantification of IRF4 and Bach2 co-binding. **c** Frequencies of differentially expressed genes bound by both Bach2 and IRF4 that are upregulated in the absence of Bach2. **d** ATAC-seq of naïve splenic CD4 T cells from *Cd4*^*Cre*^ or *Bach2*^*fl/fl*^*Cd4*^*Cre*^ mice before and after 72 h culture in Treg cell-inducing conditions. Heatmap showing *Z*-scores of read densities at peaks called differentially accessible between genotypes that also co-localize with Bach2 ChIP-seq peaks. **e** Example loci showing ATAC-seq, IRF4 ChIP-seq and Bach2 ChIP-seq tracks. Boxes indicate Bach2 binding sites with increased IRF4 occupancy and chromatin accessibility in the absence of Bach2. **f** Number of IRF4 ChIP-seq peaks called in wildtype or *Bach2*^*−/−*^ cells at sites of differentially accessible chromatin. Source data are provided as a Source Data file.

To assess the direct effects of Bach2 on IRF4 binding, we next performed Bach2 ChIP-seq on CD4 T cells cultured in Treg-inducing conditions. We identified 4927 Bach2 binding sites, associated with 3893 genes, including previously identified Bach2 targets, such as *Gadd45b* and *Prdm1* (Supplementary Fig. 6a). Motif analysis of IRF4 ChIP peaks primarily returned the AP-1 motif and the AP-1/IRF composite element (AICE), while motif analysis of Bach2 ChIP peaks identified the long half of the Maf recognition element (MARE) (Supplementary Fig. 6b). Bach2 and IRF4 co-localized at 942 sites corresponding to 864 genes, including genes differentially expressed in the absence of Bach2 in an IRF4-dependent manner such as *Il10* and *Il2ra*, which showed enhanced binding of IRF4 in the absence of Bach2 (Fig. 8a). Four hundred and eighteen sites were bound by IRF4 only in *Bach2*^*−/−*^ cells (Fig. 8b). While genes bound by IRF4 specifically in wildtype cells were not enriched among the genes upregulated in *Bach2*^*−/−*^ cells, 80.4% of those bound by IRF4 in the absence of Bach2 and 76.9% of those bound by IRF4 in both genotypes were upregulated relative to wildtype cells (Fig. 8c), suggesting that Bach2 inhibited the local binding of IRF4 to prevent inappropriate overexpression of target genes. Consistent with this idea, IRF4 binding was more pronounced at Bach2-bound sites within or adjacent to differentially expressed genes compared to sites distant to these genes (Supplementary Fig. 6c).

We next performed ATAC-seq on wildtype and Bach2-deficient CD4 T cells cultured as above. We identified 41,420 sites of open chromatin across both genotypes, about 10% (4264) of which showed differential accessibility in Bach2-deficient compared to wildtype T cells (Fig. 8d). Consistent with Bach2 regulating TCR-responsive processes, differentially accessible sites were identified in genes known to be regulated following T cell activation, including *Tcf7*, *Il7r*, *Ccr7*, and *Il2ra*, associated with effector differentiation, including *Prdm1*, *Itgae*, and *Cd44*, or with Treg cell differentiation, such as *Ctla4*, *Tigit*, *Il10*, and *Satb1* (Fig. 8e). In line with this notion, 493 differentially accessible sites were present only or showed increased chromatin accessibility in the absence of Bach2 and overlapped with IRF4 ChIP peaks (Fig. 8f), suggesting that limiting chromatin accessibility represents one of the mechanisms by which Bach2 controls the DNA binding activity of IRF4. Finally, to assess the contribution of Foxp3 to altered chromatin accessibility of Bach2-deficient T cells, we identified Treg cell-specific accessible chromatin regions by comparing naïve and activated Treg and conventional CD4 T cells by ATAC-seq analysis. This approach identified 3381 differentially accessible sites specific to Treg cells. Importantly, only 304 (6.7%) of the sites differentially accessible between Bach2-deficient and control cells were Treg-cell specific, and the overall pattern of accessibility was similar between Treg-associated and non-associated sites between genotypes (Supplementary Fig. 6d), indicating that Bach2 regulated chromatin accessibility independent of Foxp3. Together, these observations indicate that Bach2 acts to inhibit the genomic binding of IRF4 and activation of its target genes in T cells.

## Discussion

TCR signaling plays a critical role at multiple points in Treg cell differentiation and is required for the ongoing survival of mature Treg cells^17–19^. However, it has remained incompletely understood how Treg cells can be maintained in a quiescent state despite their high affinity for self-antigen and their ongoing need for TCR signaling. Here we found that Bach2 is a key regulator of Treg cell homeostasis downstream of TCR signaling, preventing premature differentiation of effector and tissue Treg cells. In addition, Bach2 is required for the development of peripherally induced Treg cells in the gastrointestinal tract. Bach2 counteracts DNA-binding activity of IRF4 and limits chromatin accessibility, thereby attenuating IRF4-dependent transcriptional programs. Thus, IRF4 and Bach2 balance TCR signaling induced transcriptional activity to maintain Treg cell homeostasis.

The TCR-induced transcription factor IRF4 plays a key role in translating TCR signaling into appropriate transcriptional programs^40^. In Treg cells, IRF4 is critical for driving the differentiation and function of fully suppressive eTreg cells^7^, and IRF4-deficient Treg cells fail to control autoimmune pathology^43^. The transcriptional activity of IRF4 is promoted by multiple other transcriptional regulators, including AP-1, NFAT, and STATs^44–47^. We found that Bach2 restrains DNA binding of IRF4 and thereby limits the activation of transcription downstream of TCR signals. Thus Bach2 is required to limit premature eTreg cell differentiation. Consistent with this model, greater numbers of Bach2-deficient Treg cells express molecules critical for Treg cell function, including IL-10, CTLA4, and TIGIT, and display reduced expression of molecules associated with naïve T cells, such as CCR7, CD62L, and TCF1. Similarly, mice with a Treg-cell specific deletion of Bach2 had substantially increased numbers of KLRG1^+^ tissue Treg cells in several non-lymphoid tissues, including the gastrointestinal tract. Strikingly, *Bach2*^*fl/fl*^*Foxp3*^*Cre*^ mice almost completely lacked RORγt^+^ pTreg cells and were largely protected from experimentally induced colitis. This is not only consistent with a critical role of IL-10 in this disease model^36,37^ but also in line with our recent observations according to which loss of RORγt^+^ pTreg cells correlated with protection from DSS-induced colitis^38^. In contrast, in adoptive transfer experiments, Bach2-deficient Treg cells showed impaired proliferative capacity and survival compared with control cells. Thus, the improved suppression mediated by Bach2-deficient Treg cells is counterbalanced by their poor maintenance.

A requirement for Bach2 to attenuate the transcriptional interpretation of TCR signals was evident in two distinct modes of Treg cell development. While Bach2 limited activation and further differentiation of thymically derived Treg cells, it was also necessary for the differentiation of peripherally induced Treg cells in the colon. Notably, the requirement for Bach2 was not related to its role in limiting expression of pro-inflammatory mediators such as IFN-γ or IL-4, or transcription factors such as GATA3 or Blimp1. Instead, we found that peripherally induced Treg cells required Bach2 to dampen TCR-induced transcriptional activity to allow Foxp3 induction. Consistent with this model, impaired Treg cell differentiation in the absence of Bach2 could be overcome by reducing antigenic stimulation, or by the additional deletion of IRF4. Indeed, pTreg cells were largely absent from the gastrointestinal tracts of Bach2-deficient mice but could be partially restored by additional IRF4 loss. Importantly, loss of Bach2 could also overcome the block in eTreg cell differentiation observed in IRF4-deficient Treg cells, indicating that Bach2 and IRF4 counteract one another during both inducible Treg cell and eTreg cell differentiation.

In line with a central role for Bach2 in transcriptional regulation in response to TCR signaling, RNA-seq showed that large numbers of genes are deregulated in the absence of Bach2 following T cell activation. Notably, the majority of the genes deregulated in TCR-stimulated *Bach2*^*−/−*^ CD4 T cells were normalized by the additional loss of IRF4, suggesting that Bach2 can inhibit the transcriptional activity of IRF4. In line with this model, ChIP-seq analysis identified close to twice as many IRF4 binding sites in *Bach2*^*−/−*^ cells compared with wildtype controls. Importantly, the IRF4-dependent rescue of the deregulated gene expression and the block in Treg cell differentiation in the absence of Bach2 was independent of Foxp3 itself. Consistent with local inhibition of IRF4 binding by Bach2, many of the sites that showed differential IRF4 binding co-localized with Bach2 binding sites. Motif analysis of our Bach2 ChIP-seq data identified the Maf recognition element (MARE), which contains an embedded AP-1 binding site (TGCTGA^G^/_C_TCAGCA), suggesting that Bach2 competes with IRF4-recruiting AP-1 complexes for genomic binding sites. This model is consistent with an emerging picture of Bach2 activity in conventional T cells, in which Bach2/BATF complexes negatively regulate the transcription of Th2 lineage effector genes, while BATF/IRF4 complexes promote transcription of these genes^48^. Similarly, Bach2 was found to limit the genomic binding of c-Jun, concomitantly inhibiting terminal differentiation of CD8 T cells^49^. On the basis of these data, we propose that Bach2 dynamically regulates the transcriptional interpretation of TCR signal strength by regulating the access of IRF4/AP-1 complexes to shared genomic binding sites. In this model, Bach2, induced by TCR signaling, binds to MARE sites and inhibits the binding of AP-1 transcription factors and the subsequent recruitment of IRF4. In the absence of Bach2, AP-1/IRF4 complex binding is increased, leading to enhanced transcriptional activity in response to TCR signals.

Interestingly, the TCR-induced kinase AKT has been identified to inactivate Bach2 by mediating its phosphorylation at Serine-535^49–51^. This provides a feedback mechanism by which the magnitude of the transcriptional response to TCR signals may be further modulated by Bach2. Notably, IRF4 loss normalized expression of the majority but not all of the genes differentially expressed in the absence of Bach2, suggesting that some MARE-binding factors may act in an IRF4-independent manner to moderate transcription. Furthermore, we observed enhanced IRF4 binding at chromatin sites ectopically open in Bach2-deficient cells that did not colocalize with Bach2 ChIP peaks. This suggests that Bach2 may regulate IRF4 binding through long-range regulation of chromatin accessibility or through regulation of expression of chromatin modifiers. Notably, our data contrast with a previously proposed model according to which conventional effector differentiation represents a competing fate that inhibits pTreg cell differentiation in the absence of Bach2^27^. Indeed, neither loss of Blimp1, nor blocking IFNγ or IL-4 signaling, or GATA3 expression, rescued pTreg differentiation in the absence of Bach2. Rather, our data indicate that modulation of TCR-induced transcription by Bach2 is central to the development of peripherally induced Treg cells and to the homeostasis of thymically derived Treg cells.

Although Bach2 is expressed by both conventional and regulatory T cells, it plays a particularly important role in Treg cells, which due to their high affinity for self-antigen consistently experience TCR signaling, leading to persistent expression of TCR-sensitive factors such as IRF4. Thus, Bach2-dependent control of TCR-induced transcriptional activity constitutes an important mechanism that maintains the quiescence of the mature Treg cell pool. Given that the same mechanism is employed to promote the development of peripherally induced Treg cells, our results show that the Bach2/AP-1/IRF4 axis constitutes a highly conserved mechanism that shapes both the thymically-derived and peripherally-derived Treg cell compartments and preserves the pool of mature Treg cells.

## Methods

### Mice

*Bach2*^*fl/fl*^ (authors), *Bach2*^*RFP*^ (authors), *Bach2*^*−/−*^ (provided by K. Igarashi), *Foxp3*^*Cre*^ (JAX 016959), *Foxp3*^*RFP*^ (JAX 008374), *Foxp3*^*DTR*^ (JAX 016958), *Cd4*^*Cre*^ (JAX 022071), *Blimp1*^*GFP*^ (authors), *Irf4*^*−/−*^ (JAX 031834), *Irf4*^*fl/fl*^ (JAX 009380), *Blimp1*^*fl/fl*^*Lck*^*Cre*^ (authors), and Smarta (JAX 030450) mice were maintained on a C57/B6 background and have been described previously^31,33,52–61^. Male and female mice were used in sex-matched experimental groups. Mice were maintained and used in accordance with the guidelines of Animal Ethics Committees of the Walter and Eliza Hall Institute of Medical Research and Peter Doherty Institute for Infection and Immunity.

### Antibodies and flow cytometry

Fluorochrome-conjugated antibodies directed against the following mouse antigens were used for analysis by flow cytometry: CD4-FITC (BD 557307, 1:400), CD4-A700 (WEHI Monoclonal Antibody Facility, 1:3200), CD8-BV650 (BioLegend 100742, 1:400), TCRb-PerCP/Cy5.5 (eBioscience 45-5961-82, 1:300), Foxp3-PerCP/Cy5.5 (eBioscience 45-5773-82, 1:300), Foxp3-E450 (eBioscience 48-5773-82, 1:300), Foxp3-PE (eBioscience 12-5773-82, 1:300), CD25-PE (BioLegend 101904, 1:400), CD25-PE/Cy7 (eBioscience 25-0251-82, 1:800), CD25-Biotin (BD 553070, 1:500), CD45.1-Biotin (eBioscience 13-0453-85, 1:500), CD45.1-FITC (BD 553775, 1:300), CD45.1-APC (BD 558701, 1:300), CD45.1-E450 (eBioscience 48-0453-82, 1:300), CD45.2-Biotin (eBioscience 13-0453-82, 1:500), CD45.2-FITC (BD 553772, 1:300), CD45.2-APC (BD 558702, 1:300), CD45.2-BUV395 (BD 564616, 1:300), CD44-APC (BD 559250, 1:400), CD44-PE/Cy7 (eBioscience 25-0441-82, 1:1600), CD44-BUV395 (BD 740215, 1:400), CD62L-APC (BD 553152, 1:300), CD62L-PE/Cy7 (eBioscience 25-0621-82, 1:1600), RFP-FITC (Abcam ab34764, 1:300), KLRG1-FITC (eBioscience 11-5893-82, 1:300), KLRG1-PE/Cy7 (eBioscience 25-5893-82, 1:300), TIGIT-E660 (eBioscience 50-9501-82, 1:200), CTLA4-APC (BioLegend 106310, 1:300), ICOS-PerCP/E710 (eBioscience 46-9942-82, 1:200), ICOS-PE (eBioscience 46-9940-82, 1:400), CCR7-APC (BioLegend 120108, 1:50), Ki67-FITC (BD 556026, 1:200), CD103-PE (eBioscience 12-1031-82, 1:300), ST2-PerCP/E710 (eBioscience 46-9333-82, 1:300), IL-10-PE/Cy7 (BioLegend 505026, 1:200), Ly6c-PE/Cy7 (eBioscience 25-5932-82, 1:300), CD11b-APC (eBioscience 17-0112-82, 1:300), RORgt-BV421 (BD 562894, 1:200), GATA3-PE (eBioscience 12-9966-42, 1:50), Helios-A647 (BioLegend 137218, 1:200), IFN-g-FITC (WEHI Monoclonal Antibody Facility, 1:1600), IL-4-APC (BD 554436, 1:200), IL-17a-PE (BD 559502, 1:200), TCR Va2-APC (eBioscience 17-5812-82, 1:400), TCR Va2-PE (eBioscience 12-5812-82, 1:1600). For intracellular staining of Bach2 reporter, cells were pre-fixed for 20 min in 4% paraformaldehyde. For chemokine receptor staining, cells were incubated at 37 °C in the presence of antibody. Brilliant stain buffer (BD) was added to antibody staining cocktails containing two or more Brilliant Violet fluorophores. Western blots were probed with anti-Histone H3 (Cell Signaling Technology #9715, 1:3000) HRP-conjugated anti-Rat IgG (Santa Cruz sc-2006, 1:3000), HRP-conjugated anti-biotin (Santa Cruz sc-57636, 1:3000) and actin detected using HRP-conjugated anti-actin (Santa Cruz sc-1616, 1:3000). A biotin-conjugated monoclonal anti-Bach2 antibody (clone 7A4, isotypes IgG2a, κ, 1:1000) was generated by immunizing rats with a purified bacterial his-tagged fusion protein containing the 58 C-terminal amino acids of mouse Bach2 and screening hybridoma supernatants with a glutathione-S-transferase (GST)-fusion protein containing the same Bach2 residues (WEHI Antibody Facility). Flow cytometry data were collected on the FACSCanto II, LSRFortessa, LSRFortessa X20 (BD) and Aurora (Cytek) platforms, and cells flow cytometrically sorted using FACSAria II, FACSAria III or FACSAria Fusion (BD) platforms. Flow cytometry data was analyzed using *FlowJo* software (FlowJo LLC).

### T cell culture

All cells were cultured in complete IMDM media. Naïve conventional CD4 T cells (CD62L+CD44-CD25-) were purified using flow cytometric sorting (sorting profile in Supplementary Fig. 7). They were then stimulated by culture in plates pre-coated with anti-CD3 (2C11, 10 μg/mL) and with soluble anti-CD28 (37.51, 2 μg/mL) (WEHI Antibody Facility). Naïve CD4 T cells from Smarta TCR-transgenic mice were purified using magnetic beads (Miltenyi) and stimulated by culture in the presence of irradiated (1000 rads) splenocytes depleted of T cells using anti-CD4 (GK1-4) and anti-CD8 (YTS-169) antibodies (WEHI Antibody Facility) and the indicated concentrations of antigenic peptide. To induce in vitro Treg cell differentiation, media was supplemented with 100 U/mL recombinant human IL-2, 0.5 ng/mL TGF-β, and unless otherwise indicated 10 μg/mL anti-IL-4 (BVD4) and 10 μg/mL anti-IFN-γ (AN18) antibodies. For Th polarization conditions, naïve CD4 T cells were isolated by magnetic beads and cultured in media supplemented with the following cytokines and antibodies Th1: 10 ng/mL IL-12 (Peprotech), 10 μg/mL anti-IL-4. Th2: 20 ng/mL IL-4 (R&D systems), 10 μg/mL anti-IFN-γ. Th17: 30 ng/mL IL-6 (Peprotech), 0.5 ng/mL TGF-β, 10 μg/mL anti-IFN-γ and anti-IL-4. Restimulation for cytokine production was performed using phorbol 12-myristate 13-acetate (PMA, 50 mg/mL) and ionomycin (0.5 mg/mL) (Sigma) in the presence of Brefeldin A and Monensin (BD Biosciences) in complete IMDM for 3 h at 37 °C. The BD Bioscience Fixation and Permeabilisation kit was used for intracellular analysis of cytokines according to manufacturer’s instructions.

### Induction of colitis with dextran sodium sulfate

To induce acute colitis, mice were provided with 1.5% dextran sodium sulfate (w/v) (MW ca 40,000; MP Biomedicals) dissolved in drinking water for 5 days, followed by 3 days with normal water. At the end of the experiment (day 8), the length of the colon was measured from the cecum to the anus. To analyze histological changes, the distal part of the colon was fixed in 10% formalin, embedded in paraffin, and cut into 2-mm sections. The colon sections were deparaffinized and stained with H&E. Fixed colon samples were scored in a blinded manner. The histological score of DSS colitis was determined as the sum of individual scores for inflammatory cell infiltrations and tissue damage as described^62^. Inflammatory cell infiltration: 1 (mild) mucosal infiltration; 2 (moderate) mucosal and submucosal infiltration; 3 (marked) transmural infiltration. Tissue damage: 1, focal erosions; 2, erosions ± focal ulcerations; 3, extended ulcerations ± granulation tissue ± pseudopolyps.

### Isolation of intestinal lamina propria lymphocytes

Peyer’s patches and visible fat were removed and the colon was opened longitudinally and cut into small pieces (<5 mm). Epithelial cells and intraepithelial lymphocytes were removed by washing with Hank’s Buffered Salt Solution (HBSS) and incubating with 5 mM EDTA for 30 min at 37 °C. The intestinal pieces were washed with RPMI and 10% FCS, and LPLs were isolated by digestion with 1 μg/mL DNase (Sigma-Aldrich) and 200 μg/ml Collagenase III (Worthington) for 40 min at 37 °C. Lamina propria lymphocytes were purified by 40–80% Percoll (GE Healthcare) gradient.

### Isolation of lymphocytes from the liver

Mice were perfused post-cull by injection of 20 mL of PBS into the left ventricle or portal vein. Livers were disaggregated through 70 μm filters and lymphocytes separated from hepatocytes by 33% Histopaque gradient.

### Inducible Treg cell depletion

Foxp3 diphtheria toxin receptor transgenic mice (C57/B6 background, described previously^33^) were injected intraperitoneally with 1 μg diphtheria toxin (CSL) one day prior to and two days following adoptive Treg cell transfer of 200,000 CD4^+^Foxp3^+^ T cells in 200 μL of PBS (sorting profile in Supplementary Fig. 7).

### Malaria infections

To prepare infectious erythrocytes, passage mice were infected with 200 μL cryo-preserved *P. chabaudi chabaudi* AS parasitised red blood cell inoculum via intravenous tail injection. When passage parasitemia reached 2–4% (typically 2–4 days post inoculation), blood was harvested and prepared for inoculation of experimental mice. Briefly, passage mice were euthanized using CO_2_ inhalation, blood was harvested via cardiac bleed and washed in media (5 IU heparin, Pfizer, NSW, Australia), 1% (w/v) penicillin/streptomycin (Gibco, Thermo Fischer, Walther, MA, USA), in RPMI. The concentration of pRBC was adjusted to 5 × 10^5^ per mL in RPMI/PS. Experimental mice were infected with 10^5^ iRBC via intravenous (i.v.) tail injection.

### RNA-seq

Flow cytometrically sorted naïve CD4 T cells were cultured in Treg cell-inducing conditions as described above. At the indicated times post culture, RNA purification was performed using the RNeasy Plus Mini kit (QIAGEN) according to the manufacturer’s instructions. cDNA libraries were generated using the TruSeq RNA Sample Preparation Kit (Illumina) following the manufacturer’s protocol. Libraries were sequenced using the Illumina NextSeq 500, producing at least 25 million paired-end 81 bp reads. In another experiment, total Treg cells were flow cytometrically sorted from the spleens of *Bach2*^*fl/fl*^*Foxp3*^*Cre*^ and *Foxp3*^*Cre*^ control mice and subjected to RNA-seq.

### ChIP-seq

Flow cytometrically sorted naïve CD4 T cells were cultured in Treg cell-inducing conditions as described above. At 72 h, cells were cross-linked by the addition of 1% formaldehyde at room temperature for 10 min and quenched with 0.25 M glycine, followed by sonication and immunoprecipitation with 10 μg of either anti-IRF4 (M-17, Santa Cruz Biotechnology, Inc.) or anti-Bach2 (7A4). DNA fragments were blunt-end ligated to Illumina adapters, amplified, and sequenced with the Illumina NextSeq 500, producing at least 30 million paired-end 81 bp reads.

### ATAC-seq

Flow cytometrically sorted naïve CD4 T cells were cultured in Treg cell-inducing conditions as described above. At 72 h, cells were counted using a haemocytmeter and 50,000 taken for processing according to the protocol described by Buenrostro et al.^63^. DNA libraries were sequenced with the Illumina NextSeq 500, producing at least 25 million paired-end 80 bp reads. To identify Treg cell specific accessible chromatin regions we sorted naïve (CD62L^+^) and activated (CD62L^-^) Treg and conventional CD4 T cells and performed ATAC-seq. Treg cell specific sites were identified by comparing Treg cells and CD4 T cells in an activation state specific manner.

### Bioinformatic analysis

All sequencing reads were mapped to the mm10 genome using Subread-1.6.0^64^, where only uniquely mapped reads were retained. The read aligner was run on either the DNA-seq mode or the RNA-seq mode, depending on the types of the samples.

### RNA-seq analysis

Genewise counts were obtained using featureCounts^65^. Only the genes that had a CPM (counts per million mapped fragments) value greater than 0.5 in at least one sample and had known symbols were used in the downstream analysis. Counts were converted to log_2_ CPM, quantile-normalized, and precision-weighted with the voom function of the limma package^66,67^. The log_2_ CPM values were then converted to log_2_ FPKM (Fragments Per Kilobase per Million) values. A linear model was fitted to each gene and empirical Bayes moderated t-statistic was used to assess differences in expression. Genes were called differentially expressed (DE) if they achieved a false discovery rate (FDR) of less than 0.05. A minimum fold change of 2 between the two conditions was also required for calling the DE genes.

### ChIP-seq analysis

Peaks were called on the mapping results using Homer-4.7^68^. The ChIP-seq samples were compared with the input samples when available, and the samples of the same type were treated as replicates. An FDR cutoff of 10^−3^ was used for calling peaks on the Bach2-ChIP samples, and 10^−8^ on the IRF4-ChIP samples. The peaks were removed from the analysis if there are no ATAC-seq peaks overlapping with it. The peaks called from the Irf4-ChIP samples were then filtered by their FPKM values in the ChIP samples and in the input samples. A peak was kept in the analysis only if it has at least 2 RPKM in all the ChIP samples where it was called, and also has at most 2 RPKM in all the corresponding input samples. A minimum fold change of 5 was also required between its minimum RPKM value in the ChIP samples and its maximum RPKM value in the corresponding input samples.

### ATAC-seq analysis

Peaks were called on the mapping results of the two samples belonging to each sample type by using Homer-4.7 at FDR cutoffs of 0.05 for the 0 h samples, and 10^−10^ for the 72 h samples. The peaks from all the samples were merged into non-overlapping regions by taking unions of the peaks, and the counts of read-pairs in the individual samples falling into the merged regions were obtained using featureCounts. Only the regions that had a CPM value greater than 8 in at least two samples were used in the downstream analysis. Counts were converted to log2 CPM, quantile-normalized, and precision-weighted with the voom function of the limma package. A linear model was fitted to each region and empirical Bayes moderated t-statistic was used to assess differences in accessibility between the different sample types. Regions were called differentially accessible (DA) if they achieved an FDR of less than 0.05.

### Statistics

If not stated otherwise an unpaired Student’s *t-*test was performed to test for statistical significance. All statistics were calculated using *Prism* software (GraphPad).

## Supplementary information

Supplementary Information

## Data Availability

Sequence data generated for this study have been deposited in the Gene Expression Omnibus (GEO) database under the primary accession code GSE126811. Raw data for figures are provided in the Source Data file. All other data are available in the manuscript and its Supplementary files or from the author upon request.

## Source data

Source Data
