# Supplementary material for: Attenuation of TCR-induced transcription by Bach2 controls regulatory T cell differentiation and homeostasis
Source: Nat Commun. 2020 Jan 14;11:252. doi: 10.1038/s41467-019-14112-2 (PMC6959360; doi:10.1038/s41467-019-14112-2)
Supplement: Supplementary file 1 — Supplementary Information [file 41467_2019_14112_MOESM1_ESM.pdf]

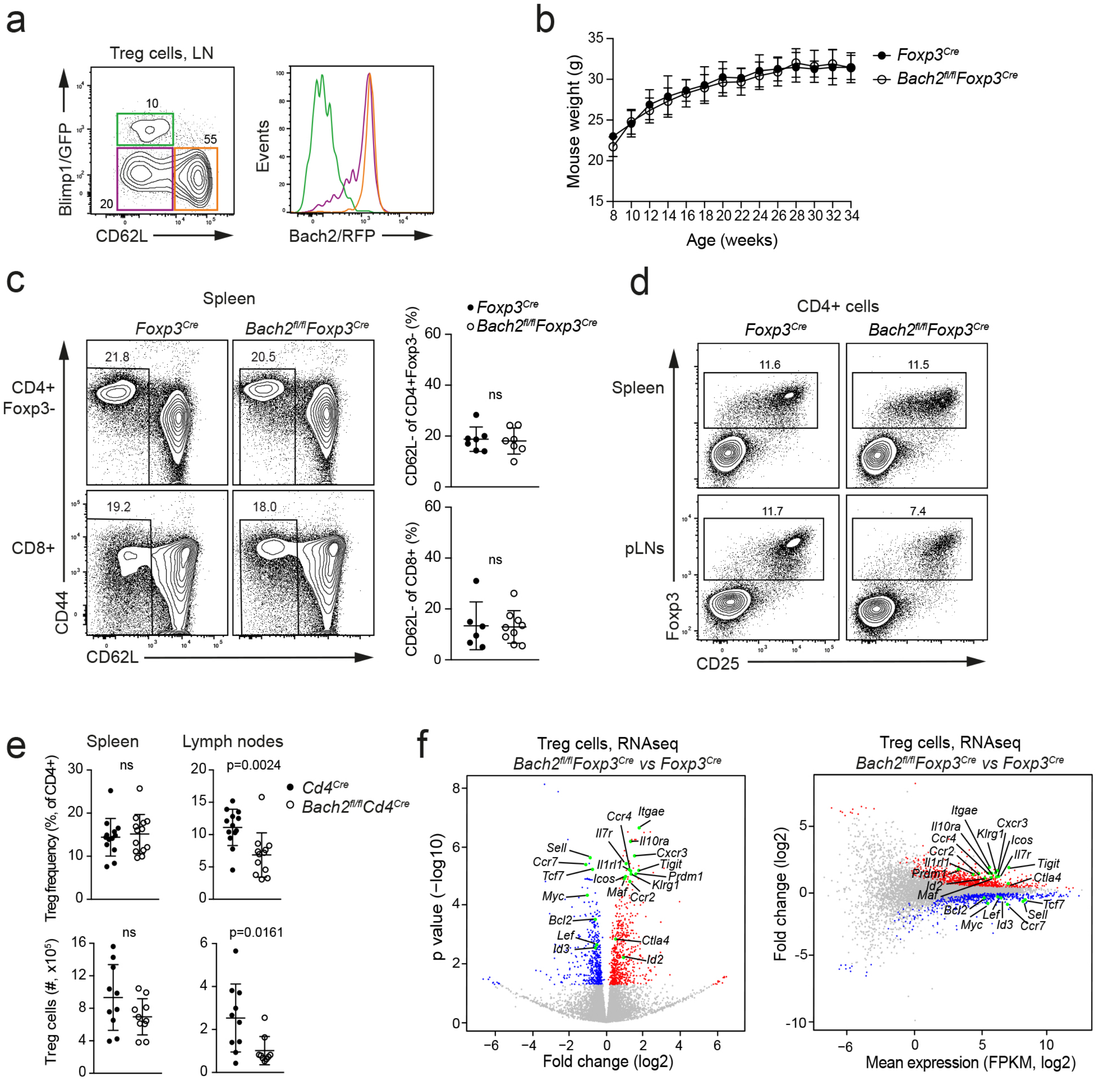

Supplementary Figure 1. *Bach2*$^{fl/fl}$*Foxp3*^{Cre} mice remain healthy. (**a**) Gating strategy for naïve (CD62L$^+$), activated (Blimp1$^-$CD62L$^-$) and Blimp1$^+$ effector Treg cells using Blimp1-GFP/Bach2-RFP double reporter mice (left) and expression of Bach2-RFP in indicated Treg cell populations (right). In this panel Treg cells are gated as CD4$^+$CD25$^+$. (**b**) Weight curve of *Foxp3*^{Cre} and *Bach2*$^{fl/fl}$*Foxp3*^{cre} mice. (**c**) Frequencies of CD62L$^-$ antigen experienced T cells in splenic CD4 and CD8 T cells of *Bach2*$^{fl/fl}$*Foxp3*^{cre} and *Foxp3*^{Cre} control mice analysed by flow cytometry. Representative flow cytometric plots (left) and quantification (right). (**d**) Representative flow cytometry plots displaying Treg cell frequencies and CD25 expression in *Bach2*$^{fl/fl}$*Cd4*^{Cre} and *Cd4*^{Cre} control spleens and pooled brachial, axial and inguinal lymph nodes. (**e**) Treg cell frequencies (upper) and numbers (lower) in the spleens and pooled brachial, axial and inguinal lymph nodes of *Bach2*$^{fl/fl}$*Cd4*^{Cre} and *Cd4*^{Cre} control mice. (**f**) Volcano (left) and mean-difference (right) plots of RNA-seq data related to **Fig. 1**e. Data representative of (a, d) of pooled from (c, e) three independent experiments. Error bars denote mean ±S.D. ns – not significant. Source data are provided as a Source Data file.

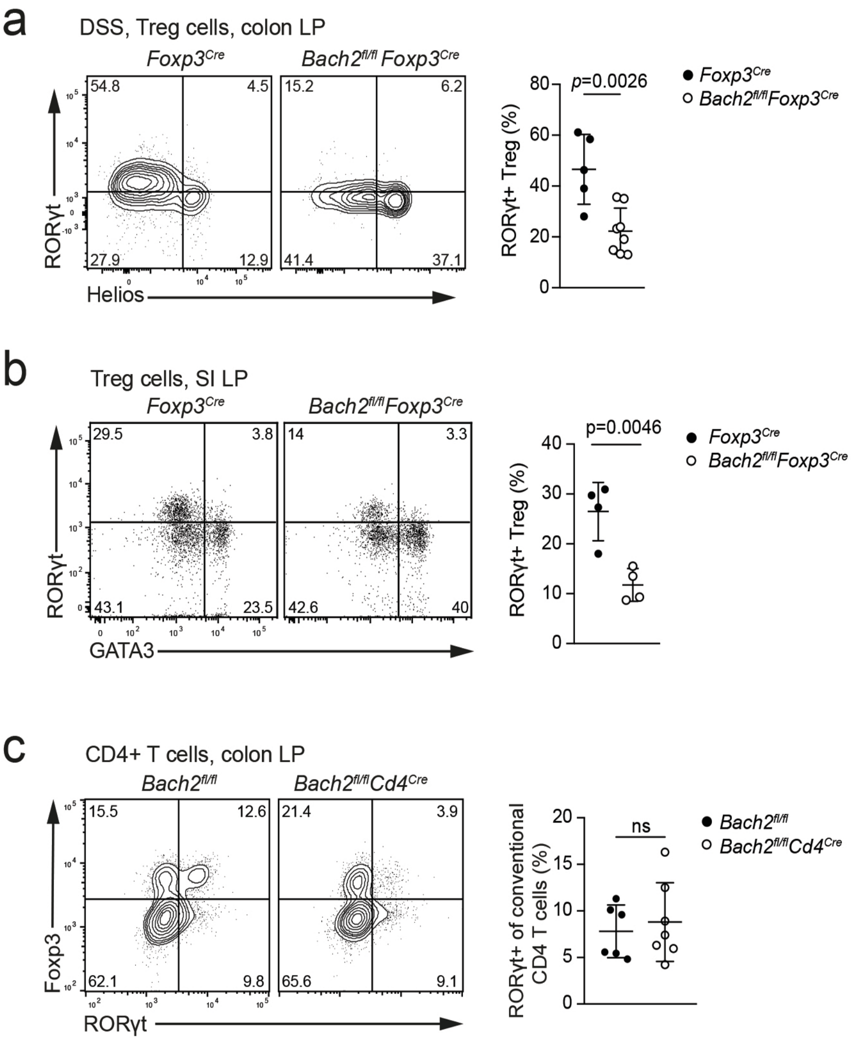

Supplementary Figure 2. Bach2 and the gastrointestinal Treg cell compartment. (**a**) Expression of RORγt and Helios in colonic Treg cells from *Bach2$^{fl/fl}$Foxp3^{Cre}* and *Foxp3^{Cre}* control mice treated with DSS as in **Fig. 3**. (**b**) Expression of RORγt and GATA3 in Treg cells in the small intestine (SI) of untreated *Bach2$^{fl/fl}$Foxp3^{Cre}* and *Foxp3^{Cre}* control mice. (**c**) Flow cytometry plots showing Foxp3 and RORγt expression by CD4^{+} cells from the colonic LP (left), and quantification of the frequency of RORγt^{+} cells among conventional (Foxp3^{-}) colonic CD4 T cells (right) in *Bach2$^{fl/fl}$Cd4^{Cre}* and *Cd4^{Cre}* control mice. Flow cytometry plots are representative, data in graphs are representative of (a, b) or pooled from (c) two independent experiments with at least 4 mice per genotype. Error bars denote mean ±S.D. ns – not significant.

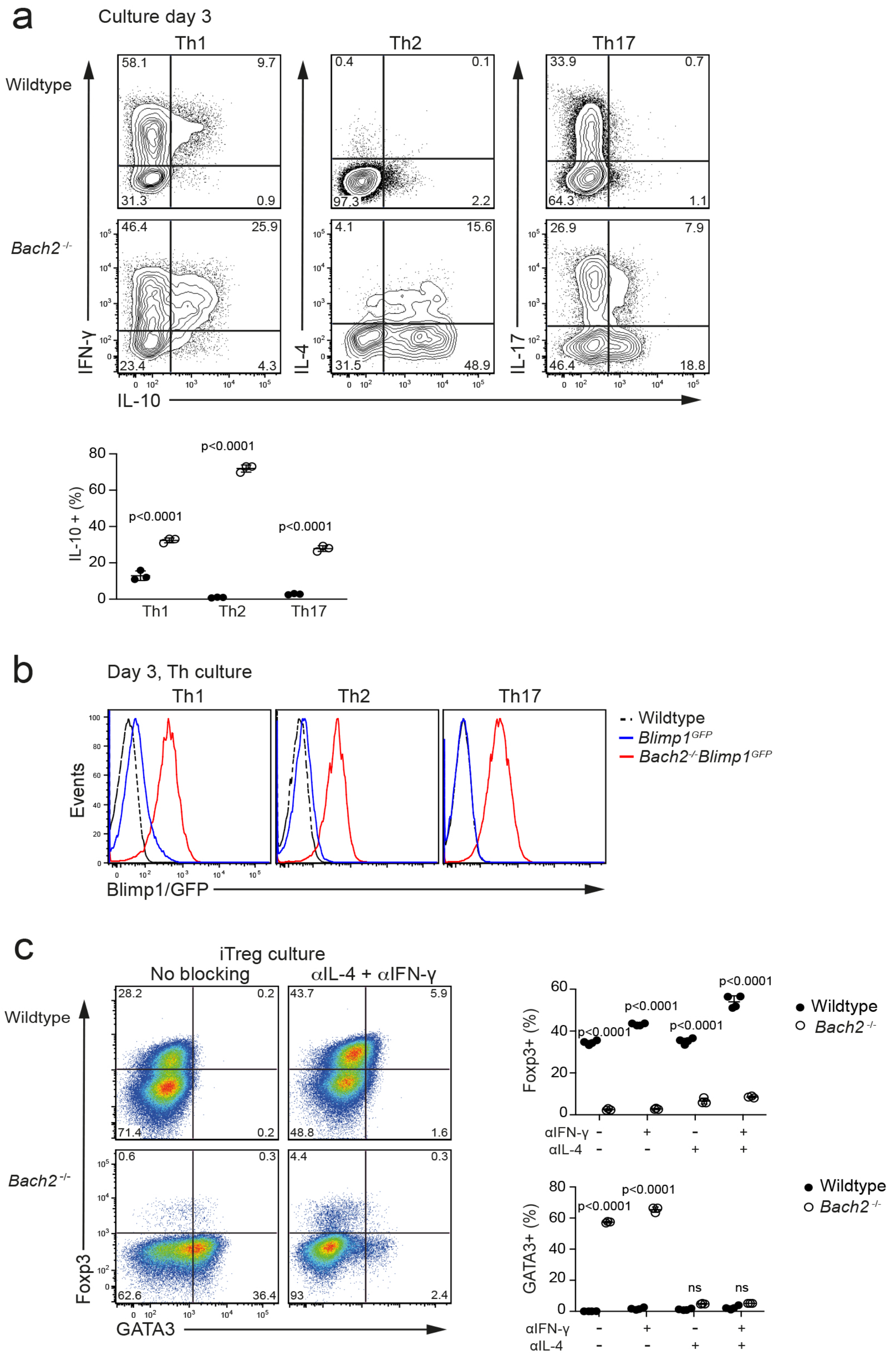

Supplementary Figure 3. Bach2-deficient T cells in *in vitro* culture. (**a, b**) Naïve CD4 T cells from *Bach2*$^{-/-}$ or wildtype control mice cultured in the indicated T helper (Th) culture conditions and expression of indicated cytokines (b) and Blimp1-GFP reporter (b) assessed by flow cytometry. (**c**) Flow cytometric analysis of Foxp3 and GATA3 expression in naïve CD4 T cells from *Bach2*$^{+/+}$ and *Bach2*$^{-/-}$ mice cultured for three days in Treg inducing conditions in the absence or presence of neutralizing anti-IL-4 or anti-IFN-γ antibodies. Representative of two independent experiments with at least five mice per genotype. Error bars denote mean ±S.D.

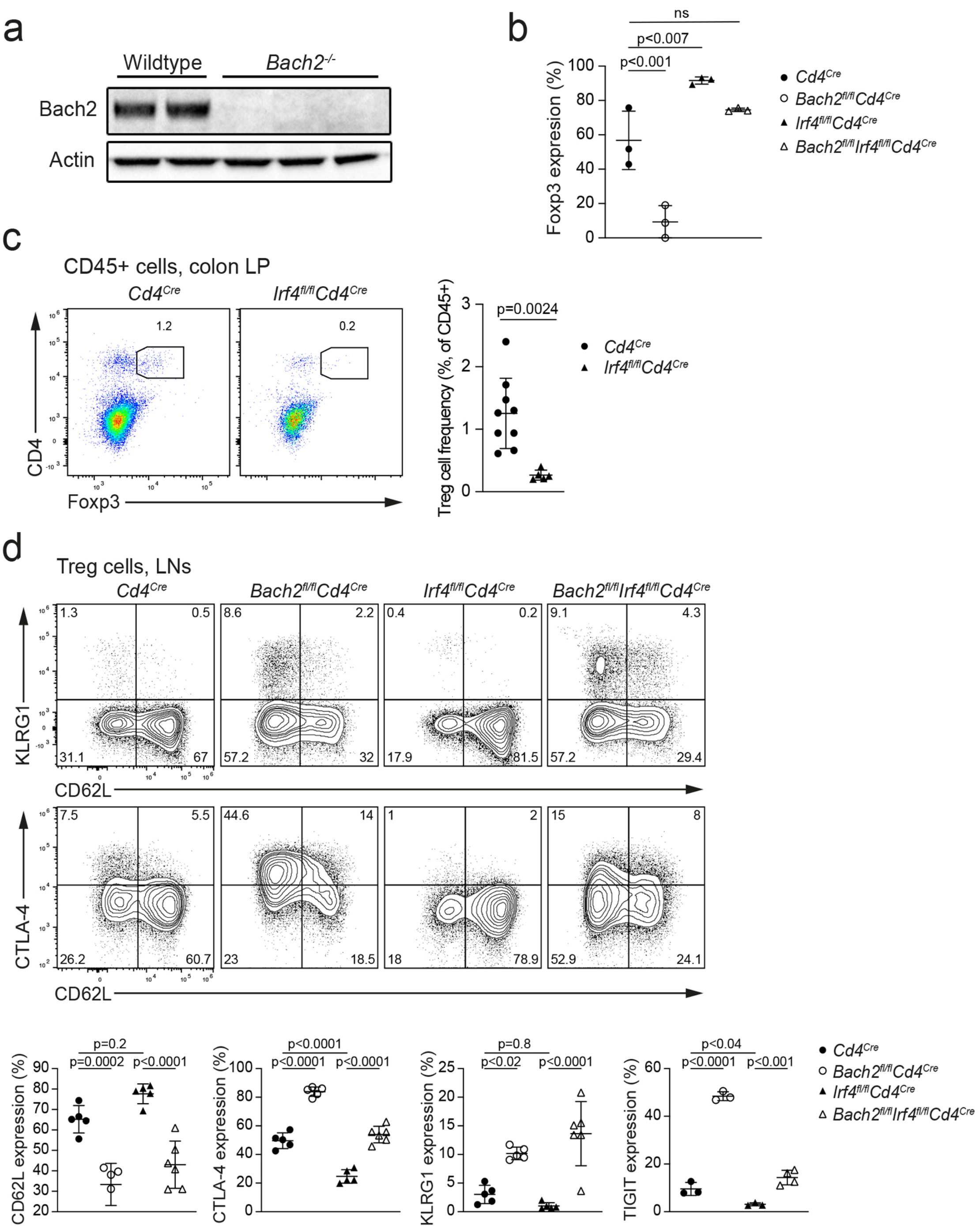

Supplementary Figure 4. Bach2 expression and IRF4-dependence of the phenotype of Bach2 deficiency. (**a**) Western blot showing Bach2 expression by naïve CD4 T cells from *Bach2*$^{-/-}$ or wildtype control mice. (**b**) Naïve CD4 T cells from *Bach2*$^{fl/fl}$*Cd4*^{Cre}, *Irf4*$^{fl/fl}$*Cd4*^{Cre}, *Bach2*$^{fl/fl}$*Irf4*$^{fl/fl}$*Cd4*^{Cre} or *Cd4*^{Cre} control mice cultured in Treg cell inducing conditions for four days and Foxp3 expression assessed by flow cytometry. Significance tested using one-way ANOVA with Dunnet's test. (**c**) Frequencies of colon lamina propria (LP) Treg cells in *Irf4*$^{fl/fl}$*Cd4*^{Cre} and *Cd4*^{Cre} control mice. (**d**) Treg cells from pooled brachial, axial and inguinal lymph nodes of *Bach2*$^{fl/fl}$*Cd4*^{Cre}, *Irf4*$^{fl/fl}$*Cd4*^{Cre}, *Bach2*$^{fl/fl}$*Irf4*$^{fl/fl}$*Cd4*^{Cre} or *Cd4*^{Cre} control mice assessed by flow cytometry for expression of activation-associated molecules. Representative plots showing expression of the indicated molecules (top) and quantification (below). Significance tested using one-way ANOVA with Tukey's test for multiple comparisons. Flow cytometry plots are representative, data in graphs are representative of (b) or pooled (c, d) from two independent experiments with at least five mice per genotype. Error bars denote mean ±S.D. ns – not significant. Source data are provided as a Source Data file.

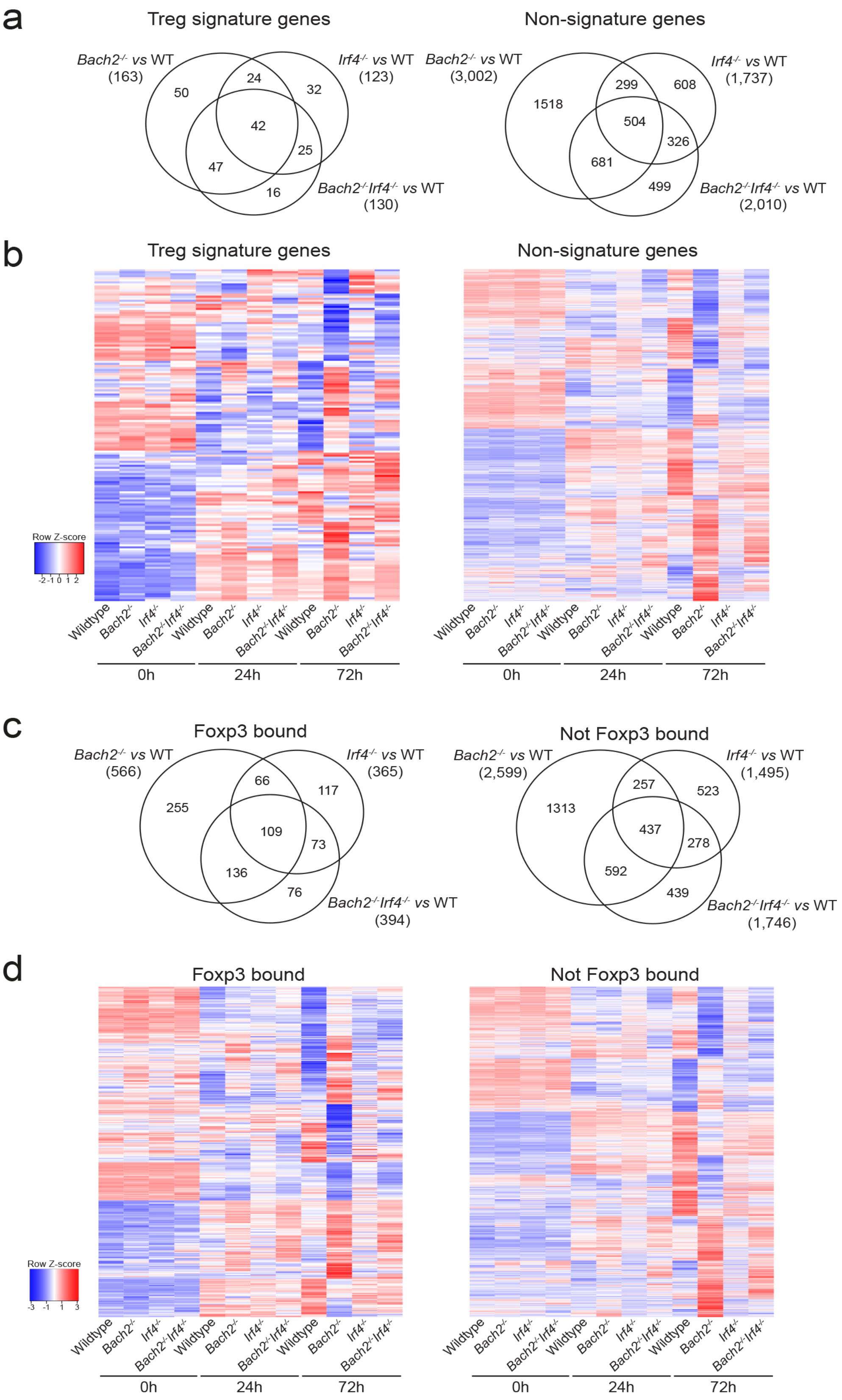

Supplementary Figure 5. Treg-cell associated genes are not uniquely differentially regulated between genotypes. (**a, b**) Differentially expressed genes in Fig. 7a stratified by status as Treg signature genes[42] (left) or non-Treg signature genes (right). Venn diagrams indicating number of genes differentially expressed in each genotype (a), heatmaps showing expression (Z-scores) of genes differentially expressed at each analysis time point (b). (**c, d**) Differentially expressed genes as in (a, b), segregated by status of genes as Foxp3 bound or not bound[11]. Source data are provided as a Source Data file.

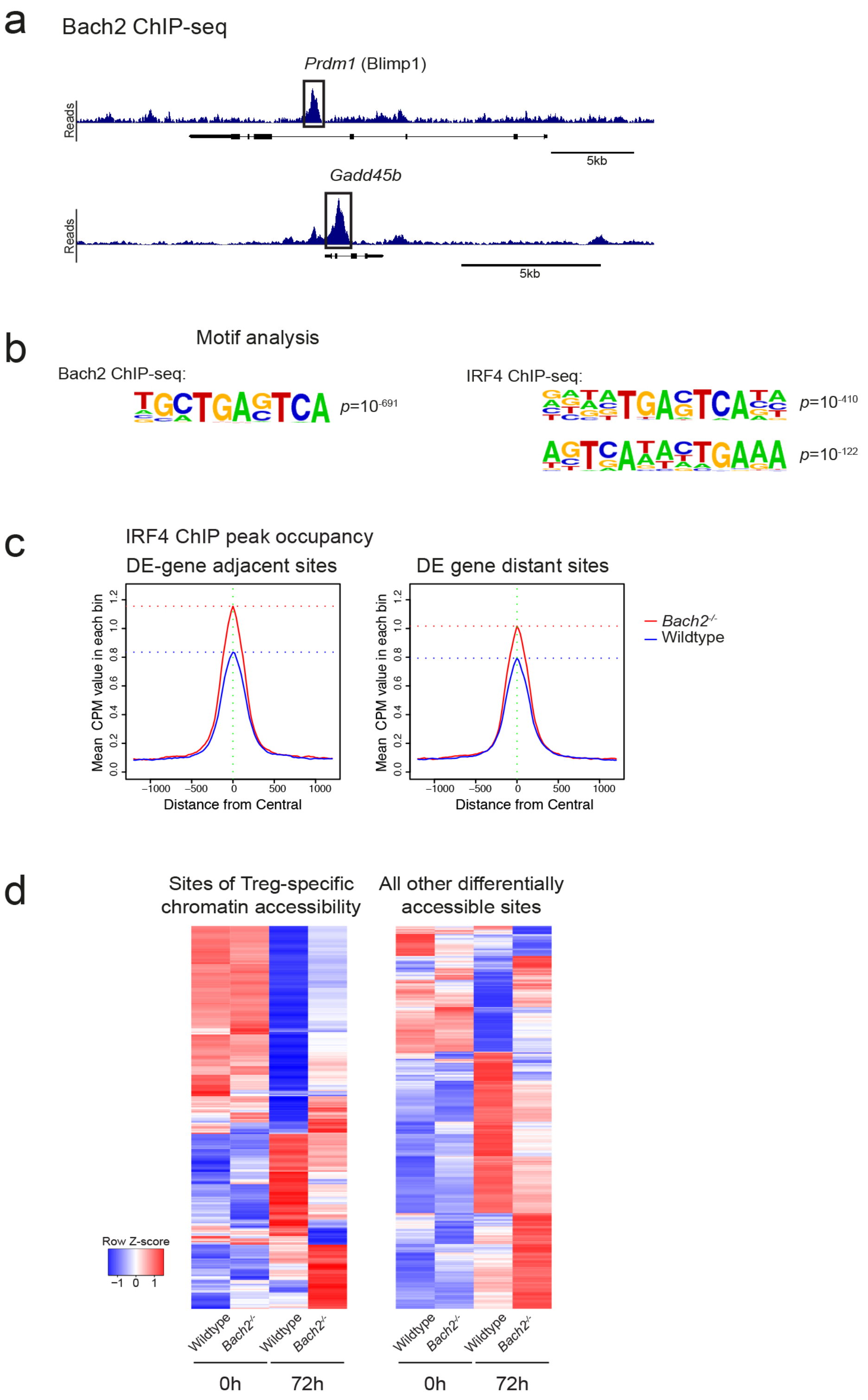

Supplementary Figure 6. Analysis of ChIP-seq and ATAC-seq data. (**a**) Example tracks showing Bach2 ChIP-seq peaks in the *Prdm1* (Blimp1) and *Gadd45b* loci. (b) High scoring motifs of IRF4 and Bach2 binding sites identified by IRF4 and Bach2 ChIP-seq performed on wildtype CD4 T cells cultured in Treg cell inducing conditions. (**c**) Mean occupancy of IRF4 at Bach2 binding sites adjacent (left) or distant (right) to genes differentially expressed in wildtype vs *Bach2*$^{-/-}$ CD4 T cells cultured in Treg cell inducing conditions. (**d**) ATAC-seq of splenic CD4 T cells from *Cd4*^{Cre} or *Bach2*$^{fl/fl}$*Cd4*^{Cre} mice before and after 72h culture in Treg cell-inducing conditions. Heatmap showing Z-scores of read densities at peaks called differentially accessible between genotypes, segregated by status as a Treg cell associated differentially accessible region. Source data are provided as a Source Data file.

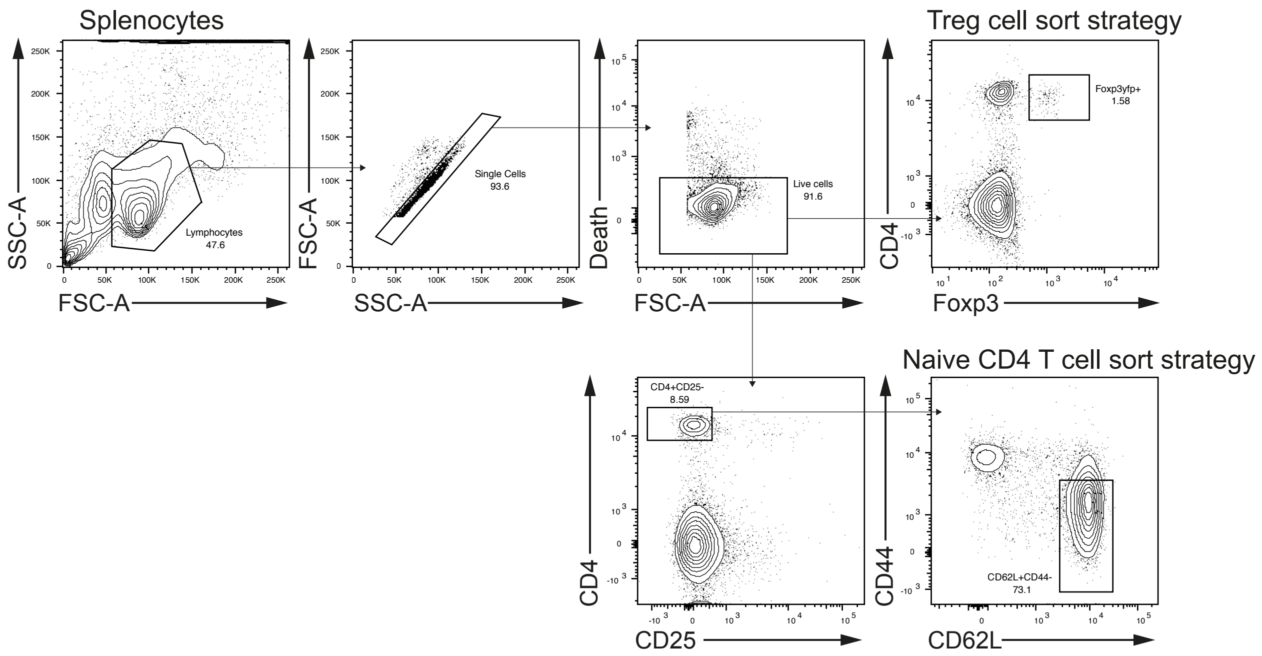

Supplementary Figure 7. Gating strategies used for flow cytometric isolation of Treg and naïve conventional T cells.
